# AURKB promotes immunogenicity and immune infiltration in clear cell renal cell carcinoma

**DOI:** 10.1007/s12672-024-01141-7

**Published:** 2024-07-16

**Authors:** Weihao Liu, Ying Liu, Shisheng Chen, Jialiang Hui, Shuhua He

**Affiliations:** 1 Department of Urology, Nanfang Hospital, Southern Medical University, Guangzhou, 510515 China; 2 Department of Oncology, Huadu District People’s Hospital of Guangzhou, Guangzhou, 510810 Guangdong China; 3 Department of Urology, Dongguan Tungwah Hospital, Dongguan, 523110 Guangdong China; 4 Department of Organ Transplantation, Nanfang Hospital, Southern Medical University, Guangzhou, 510515 Guangdong China

**Keywords:** Chromatin regulators, ccRCC, Prognostic model, Immunogenicity, TICs, Immune therapy

## Abstract

**Background:**

Chromatin regulators (CRs) are capable of causing epigenetic alterations, which are significant features of cancer. However, the function of CRs in controlling Clear Cell Renal Cell Carcinoma (ccRCC) is not well understood. This research aims to discover a CRs prognostic signature in ccRCC and to elucidate the roles of CRs-related genes in tumor microenvironment (TME).

**Methods:**

Expression profiles and relevant clinical annotations were retrieved from the Cancer Genome Atlas (TCGA) and UCSC Xena platform for progression-free survival (PFS) data. The R package "limma" was used to identify differentially expressed CRs. A predictive model based on five CRs was developed using LASSO-Cox analysis. The model's predictive power and applicability were validated using K-M curves, ROC curves, nomograms, comparisons with other models, stratified survival analyses, and validation with the ICGC cohort. GO and GSEA analyses were performed to investigate mechanisms differentiating low and high riskScore groups. Immunogenicity was assessed using Tumor Mutational Burden (TMB), immune cell infiltrations were inferred, and immunotherapy was evaluated using immunophenogram analysis and the expression patterns of human leukocyte antigen (HLA) and checkpoint genes. Differentially expressed CRs (DECRs) between low and high riskScore groups were identified using log2|FC|> 1 and FDR < 0.05. AURKB, one of the high-risk DECRs and a component of our prognostic model, was selected for further analysis.

**Results:**

We constructed a 5 CRs signature, which demonstrated a strong capacity to predict survival and greater applicability in ccRCC. Elevated immunogenicity and immune infiltration in the high riskScore group were associated with poor prognosis. Immunotherapy was more effective in the high riskScore group, and certain chemotherapy medications, including cisplatin, docetaxel, bleomycin, and axitinib, had lower IC50 values. Our research shows that AURKB is critical for the immunogenicity and immune infiltration of the high riskScore group.

**Conclusion:**

Our study produced a reliable prognostic prediction model using only 5 CRs. We found that AURKB promotes immunogenicity and immune infiltration. This research provides crucial support for the development of prognostic biomarkers and treatment strategies for ccRCC.

**Supplementary Information:**

The online version contains supplementary material available at 10.1007/s12672-024-01141-7.

## Background

In 2020, there were 431,280 new kidney tumor cases reported [1], and over 70% to 80% of these cases are clear cell renal cell carcinoma (ccRCC) [2–5]. ccRCC is the most predominant type of renal cell carcinoma [6]. For individuals with ccRCC, less than 70% of patients survive beyond 5 years after diagnosis [7]. A dismal prognosis and metastasis status were present in about one-third of these newly diagnosed patients [8]. The metastatic type of ccRCC is consistently linked to a high mortality rate [9, 10]. Therefore, there is a pressing need to create more effective prognostic models and identify target genes given the significant incidence and mortality of ccRCC.

Chromatin regulators (CRs) are the integral regulatory elements in the field of epigenetics, which studies the control of gene expression without changes to DNA sequence [11]. CRs, depending on their contribution to epigenetics, are classified into domains such as DNA methylation, histone-modifying enzymes, and chromatin remodelers [12]. Aberrant expression of CRs has been linked to several processes including inflammation [13], apoptosis [14], autophagy [15], and proliferation [16]. This implies that CR dysregulation may contribute to diverse pathological conditions, including neoplasm. Researchers have used bioinformatics analysis in recent years to identify crucial prognostic genes for ccRCC. However, CRs, a crucial aspect of epigenetics, have not received the same level of attention.

In this study, we explored the CR signature and assessed several key genes to clarify the molecular basis of ccRCC. This study aims to propose new methods for treatment decisions and prognosis prediction.

## Materials and methods

### Collecting data and screening CRs associated with progression

Transcriptome profiles of 524 individuals with ccRCC and 72 nontumor samples were collected online by the TCGA project [70]. Clinical information came from the TCGA project, and another data source was UCSC Xena [71]. From previous topic research, 870 Chromatin regulators (CRs) were identified [57]. We also downloaded the ICGC KIRC-US cohort consisting of 522 ccRCC tissues from a public database (The Cancer Genome Collaboratory, ICGC) [72] for the model’s external validation. To normalize these mRNA expression data, the relevant R package was employed. To screen for differentially expressed genes, the "limma" package was adopted using the criterion of log2|FC|> 1 and a false discovery rate (FDR) < 0.05. We used to select differentially expressed CRs having prognostic significance by the univariate Cox regression analysis (uni-Cox). Prognostic-relevant clusters with CRs associated with progression were identified using non-negative matrix factorization (NMF), and an optimal k-value factorization was chosen when the cophenetic correlation coefficient began to fall. The prognoses of two clusters, predicted by the Kaplan–Meier analysis, were revealed. Then, we selected CRs when log2|FC| was larger than 1 and the FDR was less than 0.05 which were expressed differentially in the two clusters. With a cutoff of 0.2, the correlation network of two clusters’ CRs with differential expression was mapped by the package "igraph".

### Constructing the prognostic risk model

Using the “caret” package, a 1:1 ratio was used to randomly divide the sample from the TCGA dataset into the training and testing set. To verify the signatures of the CRs, both the testing set and the whole set were employed, which were first created using the training set. By performing univariate Cox regression analysis on those CRs associated with progression, prognosis-related CRs were shown on forest plots (p < 0.05). Additionally, we mapped these CRs using the packages of “pheatmap,” “reshape2,” “limma,” and “ggpubr”. By using the LASSO Cox regression algorithm, it was possible to identify the ideal set of prognostic CRs (with the penalty parameter generated from tenfold cross-validation). The risk model was subsequently developed using multivariate Cox regression by analyzing the obtained prognostic CRs. Meanwhile, we determined the riskScore for each sample using the equation: riskScore = ∑ni = Coefi*xi, (Coefi acts for the risk coefficient, and xi for each gene’s expression).

### Evaluating this prognostic risk model’s predictive performance

Based on the median riskScores, two groups of ccRCC samples were created. A risk curve and a survival status chart were also made to show how the model's sample population was distributed. Principal component analysis (PCA) using the “scatterplot3d” package was performed for the purpose of visualizing the spatial distribution of samples. In order to determine whether there were any variations in the groups of ccRCC patients’ overall survival rate and progression free survival rate, the Kaplan Meier (K-M) curve was used. Univariate and multivariate Cox regression were carried out using the R packages “ggupbr,” “limma,” and “ComplexHeatmap” to verify the risk models' clinical validity. Through the use of receiver operating characteristic (ROC) analysis, the model's predictive ability was assessed. Each independent prognostic variable was used to construct a nomogram using R’s “rms” package. An examination of the calibration plot curve was done to see if the actual and expected survival rates were compatible. We used the packages “limma,” “survival,” “survminer,” “timeROC,” “survcomp,” “ggplot2,” and “ggpubr” to develop ROC curves and determine the area under the curve’s (AUC) value in order to evaluate the model’s accuracy. More convincing support for the model’s accuracy is derived from comparison to other published and validated models using the consistency index (C-index) [58–61]. The band diagram was displayed with the following marks: 0.001 = ***, 0.01 = ** *, and 0.05 = * to indicate if our risk model and clinicopathological variables were associated using the X2 test. It was also done to compare riskScores for various clinicopathological characteristics with the use of the Wilcoxon rank-sum test. Stratified survival studies based on the R packages “survminer” and “survival” were used to evaluate the model’s applicability.

### GO and GSEA analysis

Cellular Components (CC), Biologic Process (BP), Molecular Function (MF) of the Gene Ontology (GO) were examined with the use of the “ClusterProfiler” package. Gene set enrichment analysis (GSEA) was used to look for the underlying signaling pathway. P value < 0.05 and FDR < 25% were the criteria for defining statistical significance.

### Assessment of tumor immunogenicity

We retrieved the somatic mutation data using the Pearl programming language after getting it from the TCGA website. The top 15 frequently mutated genes were then analyzed using the “maftools” package. After reviewing and integrating the TCGA data, we assessed the difference and relationship between the three (the Tumor Mutational Burden (TMB), riskScore and survival rates).

### Assessment of immune microenvironment and immune infiltration

The ESTIMATE method was used to determine the percentages of tumour, immune, and stromal cells as indicated by TumorPurity, ImmuneScore, StromalScore, and ESTIMATEScore. Seven different methods were employed to examine the immune infiltration characteristics of the ccRCC samples (MCPCOUNTER, TIMER, CIBERSORT-ABS, XCELL, QUANTISEQ, EPIC, and CIBERSORT) [62–69]. Bubble plots were used to display immune infiltrating cell composition differences between the two riskScore groups using the Wilcoxon rank-sum test and R packages of “ggplot2, “ “limma, “ “tidyverse, “ “ggtext,” “scales”. The effect of immune cell infiltration on the prognosis was also assessed using the CIBERSORT algorithm. The LM22 data set defined 22 immune cell subsets, and it was retrieved via the CIBERSORT web page [73]. Next, immune-related functions and infiltrating immune cells were further evaluated by the single-example GSEA (ssGSEA) scoring method using the “GSVA,” “GSEABase,” and “limma” packages.

### Immune therapy and drug sensitivity analysis

We evaluated immune checkpoint activity and HLA gene expression, plotted them in box plots, and used this information to predict the efficacy of immuno treatment. The data of the TCGA KIRC samples’ Immunophenoscore (IPS) is retrieved and downloaded from the Cancer Immunome Atlas (https://tcia.at/home). A lower IPS is generally considered equivalent to a poorer immunotherapy response. The effectiveness of immunotherapeutic agents (PD1 and CTLA4 monoclonal antibodies) was predicted using IPS analysis. Conjoint analysis of the transcriptome data and FDA-certified drug sensitivity-related data, which were derived from browsing and filtering the CellMiner database (https://discover.nci.nih.gov/cellminer/), was utilised to analyse how the prognostic model's CR genes affect medication sensitivity and resistance. The Pearson correlation test was used to look at how the expression of model genes and drug sensitivity are related.

### Assessment of AURKB

After intersection of differentially expressed CRs (DECRs), utilizing log2| FC|> 1 and FDR < 0.05 as cutoff criteria, with model genes, we selected AURKB as the target gene. Kaplan–Meier (KM) survival analysis was performed to evaluate the relationship between AURKB expression and overall survival (OS). Immune cell infiltration levels were estimated using the aforementioned methods. Comprehensive Analysis on Multi-Omics of Immunotherapy in Pan-Cancer (CAMOIP) (https://www.camoip.net) was used to analyze TMB and Neoantigen Loads.

### Statistics analysis

R (version 4.2.1) was implemented to process the statistical analysis. To aid in determining the differences between two groups, a comparison analysis is performed by the use of the Wilcoxon test. P-value < 0.05 is regarded as statistically significant.

## Results

### Identifying CRs associated with progression in ccRCC

**Fig. 1:**
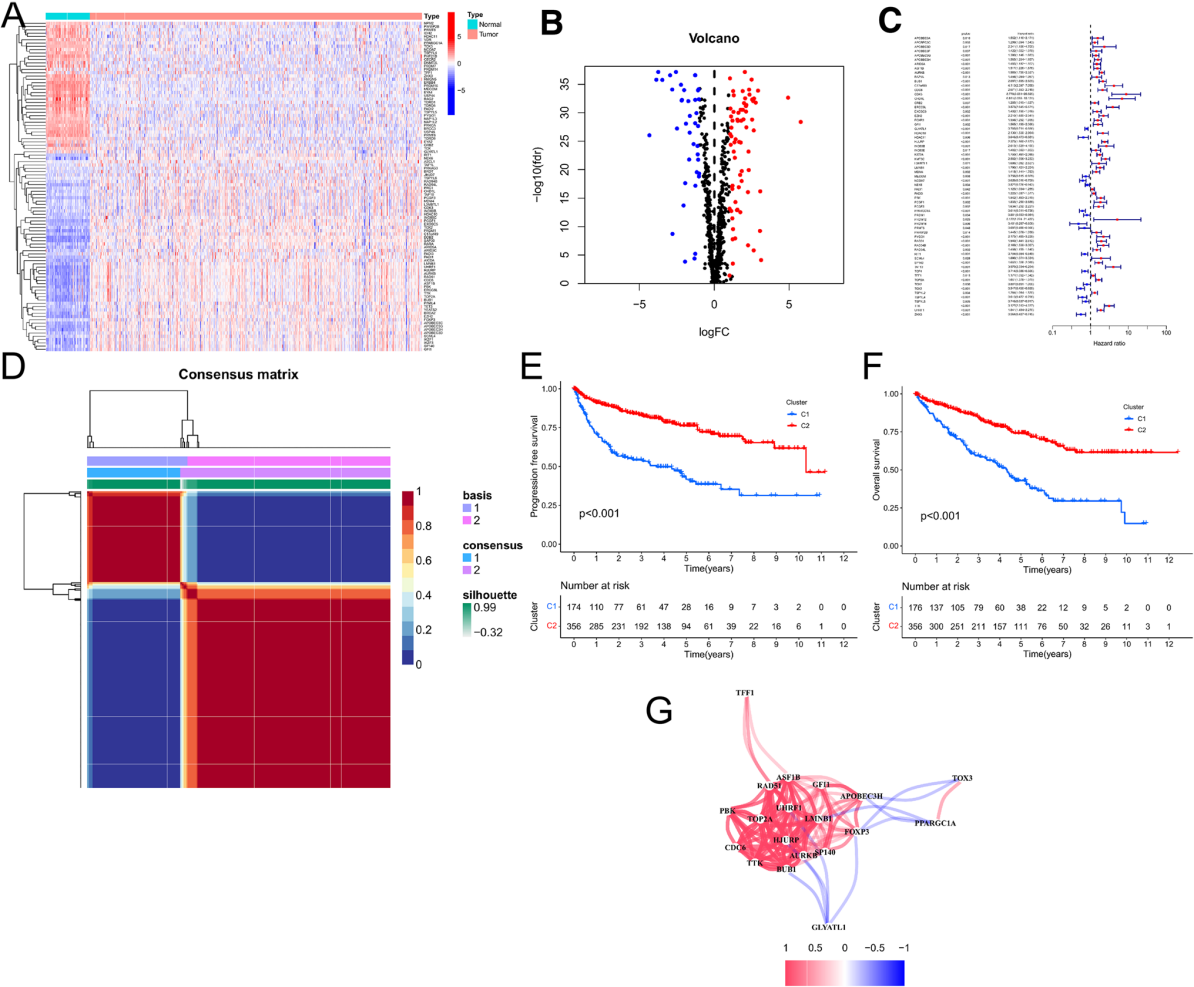
Screening of CRs associated with progression in ccRCC. **A** Heatmap showed differentially expressed CRs. **B** Volcano diagram of CRs that displayed abnormal expression in ccRCC and normal tissue specimens. Red dots: up-regulation and blue dots: down-regulation. **C **Identification of prognostic CRs by univariate Cox regression analysis. **D **ccRCC samples were clustered by nonnegative matrix factorization (NMF) method. **E**–**F** Kaplan–Meier survival curves of patients with PFS and OS in the two subclasses. **G** Co-expression networks of 20 differentially expressed CRs

We discovered 111 chromatin regulators with differential expression, of which 40 showed downregulation and 71 showed upregulation based on a comparison of tumor and normal samples (Fig. 1A–B). To identify CRs related to prognosis, we performed a univariate Cox regression analysis on the differentially expressed CRs in the TCGA-KIRC cohort. This analysis revealed that 65 of these CRs had significant prognostic value (Fig. 1C), indicating their potential roles in tumor emergence and development.

Next, we applied Non-negative Matrix Factorization (NMF) on the 65-gene panel to classify the CRs associated with malignant transformation and progression in tumor samples. Based on cophenetic correlation coefficients and consensus maps, we determined that the optimal number of clusters was k = 2 (Fig. 1D and Fig. S1). This classification divided the ccRCC cohort samples from TCGA-KIRC into two subgroups (C1, n = 176; C2, n = 356). Kaplan–Meier survival analysis showed that the C1 subgroup had a poorer prognosis compared to the C2 subgroup (Fig. 1E–F), indicating heterogeneity in the tumor samples.

To further clarify the prognosis-related CR genes, we identified differentially expressed genes between the two subgroups (C1 vs. C2) using log2|FC|> 1 and FDR < 0.05 as cutoff values. This analysis resulted in 20 differentially expressed CRs (Fig. 1G), whose gene co-expression networks were also mapped. These 20 CRs were thus associated with tumor development and progression, highlighting their potential importance in ccRCC prognosis.

### Constructing the prognostic risk model

**Fig. 2:**
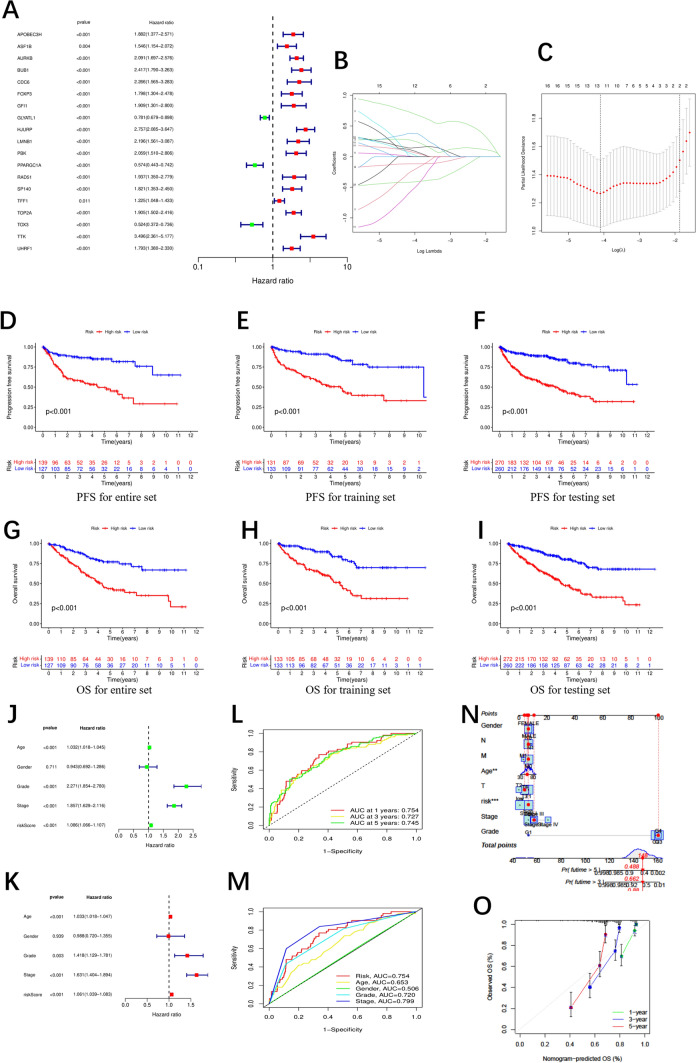
Construction and Evaluation of prognostic model of CRs. **A** Univariate Cox regression analysis of prognostic CRs in the training group. **B**, **C** cvfit and lambda curves showing the least absolute shrinkage and selection operator (LASSO) regression was performed with the minimum criteria. **D**–**I** Kaplan–Meier survival curves of patients with PFS and OS in the entire, training, and testing sets, respectively. **J**–**K** Uni-Cox and multi-Cox analyses of clinicopathologic factors and risk score with overall survival. **L** 1-, 3-, and 5 year ROC curves of the whole group. **M** 1-year ROC curves of curves for the prognostic risk model and clinicopathological characteristics. **N** Nomogram for predicting overall survival. € 1-, 3-, and 5-year overall survival of calibration curves. **O** Calibration plots of the nomograms in terms of the agreement between nomogram‐predicted and observed 1‐year survival outcomes. The 45°dashed line represented the ideal observation. The red line represented the actual prediction of the model

In order to assess their predictive potential, the ccRCC cohort from the TCGA-KIRC project was separated into two groups by simple random sampling: a group for training and a group for testing. The training group was next used as a source of training set data for building the prognostic risk model. To further screen for prognostic genes, we performed univariate Cox regression analysis on the aforementioned 20 CRs in the training group and found that 19 of the 20 CRs previously mentioned were associated with prognosis (Fig. 2A). We employed the LASSO (Least Absolute Shrinkage and Selection Operator) algorithm for variable selection. Specifically, we input the training set data into the LASSO algorithm, and after multiple iterations and parameter adjustments, 5 CRs with significant impact on the predictive model were selected. So, a model for predicting prognosis based solely on 5 CRs was developed. Figure 2B, C displays the lambda curve and cvfit. Then, 5 CRs (ASF1B, AURKB, HJURP, RAD51, and TOX3) were identified. The following is how the riskScore was calculated: riskScore = (-0.576285272 * ASF1B exp.) + (0.7782031 * AURKB exp.) + (1.195239395 * HJURP exp.) + (-0.916469765 * RAD51 exp.) + (-0.390179345 * TOX3 exp.). The higher riskScore, the greater the risk of a poor prognosis. A high riskScore group, also called a high-risk group, and a low riskScore group, also called a low-risk group, were distinguished by using the median riskScore as a guide.

According to the Chi-square test, the training and testing groups did not significantly differ in any of the clinical features (p > 0.05; Supplementary Table 1). The differences in five CRs’ gene expression patterns between the group with a low riskScore and the group with a high riskScore were shown using a heatmap. The protective CR's expression (TOX3) was downregulated in the group with high riskScore, but relatively the risk CRs (ASF1B, AURKB, HJURP, and RAD51) were upregulated (Fig.S2D-F). The distribution of patient risk levels in the two groups was shown in risk curves (Fig. S2G–I). Survival status & survival time-distribution maps are presented in Fig. S2J–L. All in all, we constructed a prognostic risk model using 5 CRs (ASF1B, AURKB, HJURP, RAD51, and TOX3).

### Evaluating this prognostic risk model’s predictive performance

To assess the distributional patterns between the two risk groups, the PCA algorithm was utilized. It was proven that, in contrast to the patterns of expression of all genes (Fig. S2A) as well as all CRs (Fig. S2B), our prognostic risk model was the most effective at separating ccRCC patients into the two risk groups defined above (Fig. S2C). The result that the PFS and OS rates were lower in the group with a high riskScore than in the group with a low riskScore could be derived from Kaplan–Meier survival analysis (Fig. 2D–I). Age, grade, stage, and risk model all demonstrated a positive link with ccRCC prognoses, as shown by univariate cox regression studies (Fig. 2J). This suggested that the aforementioned variables could affect patients' clinical outcomes. We subsequently examined the model's potential as a novel independent prognostic factor, and we found that it was capable of independently predicting a poor prognosis for ccRCC by the method of multivariate Cox regression (Fig. 2K). A time-dependent ROC curve study proved that it was practicable for the risk model to accurately forecast outcomes. Areas under the ROC curve (AUCs) was 0.754 at one year. At three years, the ROC curves had an AUC of 0.727. Five years into ROC curves, the AUC was 0.745. (Fig. 2L). The strong predictive accuracy of this signature in comparison to other clinicopathological variables was further validated by the ROC curve, showing that our model’s prognostic accuracy is only second to stage (Fig. 2M). Furthermore, risk scores and clinical-pathological factors were created to provide predictions of the 1-, 3-, and 5 year OS rates (Fig. 2N). Calibration curves demonstrated good agreement when comparing predicted overall survival rates based on this prognostic risk model to actual overall survival rates (Fig. 2O). Additionally, we performed a direct comparison of our model with several clinical prognostic models, showing that our model had the best predictive performance (Fig. S3). These results all demonstrated the risk model had a powerful capacity for survival prediction for ccRCC.

When developing a prognostic model, practical applicability should be considered. The difference in the TNM, clinical stage and grade was statistically significant (P < 0.001) through the use of Wilcoxon rank-sum test (Fig. S4A–E). Despite the fact that the gender group's riskScore did not differ significantly, the P value was close to 0.05(Fig.S4F) and the male had a higher riskScore which consisted of a higher incidence among men [1]. We inferred that the riskScore score would show a significant difference between gender groups with larger sample sizes. We also observed that the riskScore increased as the tumor became more aggressive. The outstanding applicability of the prognostic model was subsequently evaluated using stratified survival analyses. This step aims to assess the efficacy and applicability of the model across various subgroups. Stratified analysis further validates the robustness and predictive power of the model within different clinical characteristic contexts. Subgroups were separated based on factors such as grade (G1&2/G3&4), gender (female/male), age (≤ / > 65), N (N0/N1), clinical stage (I&II/III&IV), M (M0/M1), and T (T1&2/T3&4) in order to evaluate the model's predictive power in subgroups with different medical characteristics. As illustrated in Fig. S5, the data indicated that the proposed model is most likely appropriate for these clinical groups, except for stage N1, owing to the small number of samples. Finally, to further validate the prognostic performance, the same model was built using an ICGC dataset (Fig. S4G–K). The results of the survival analysis done on the ICGC dataset were consistent with our findings.

All in all, our model was a prognostic factor for independently predicting patient survival, with high predictive power and broad application based on the above-mentioned results.

### Exploring the potential mechanisms by GO and GSEA analysis

**Fig. 3:**
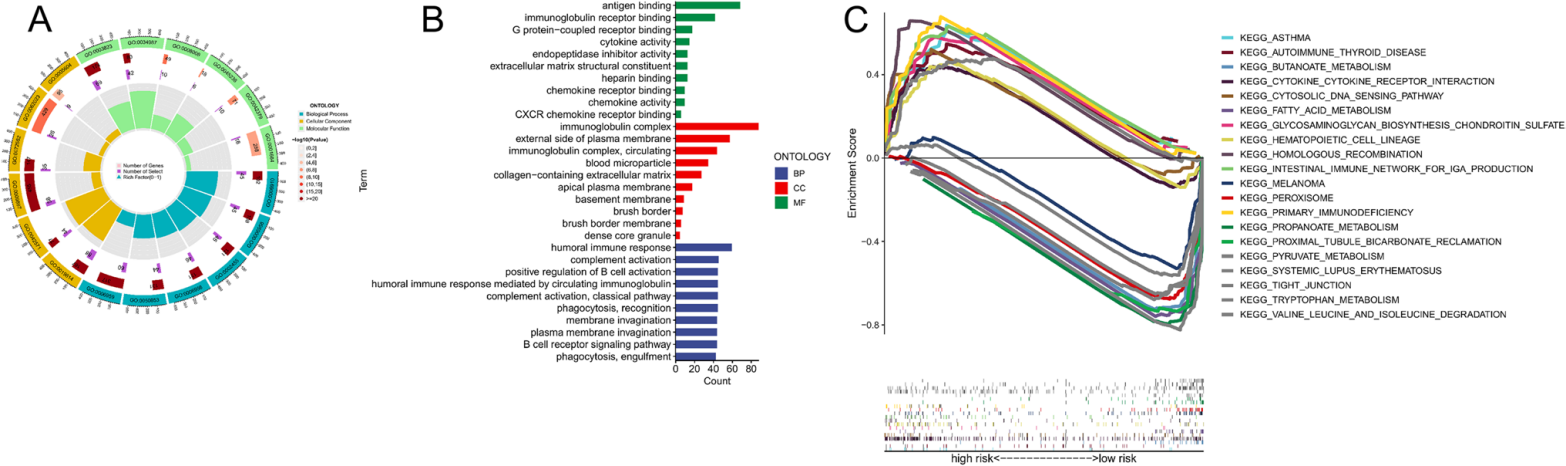
GO and GSEA analysis. **A**–**B** GO analysis showing many immune-related biological processes were enriched. **C** GSEA showing significant enrichment of immune-related pathways in the group with high riskScore

By using GO and GSEA analysis, we distinguished the biological mechanisms behind the high-riskScore and low-riskScore groups, attempting to comprehend why the group with a high riskScore had a poor prognosis. The genes used for GO enrichment and KEGG enrichment analysis are the differentially expressed genes between the low riskScore and high riskScore groups (log2|FC|> 1 and FDR < 0.05). The GO analysis showed these findings: the biological mechanisms among the groups with high and low riskScore were distinct. The group with a high riskScore, in contrast to the group with a low riskScore, had immune-related activities such as antigen binding, immunoglobulin receptor binding, immunoglobulin complex, humoral immune response, and so on (Fig. 3A–B). Further GSEA analysis revealed that half of the 10 most significantly enriched pathways in the group of ccRCC patients with a high riskScore were immune-related pathways (Fig. 3C). Additionally, numerous metabolic and tumor-associated signaling pathways existed in the group with a low riskScore, including proximal tubule bicarbonate reclamation, propanoate metabolism, and tight junction (Fig. 3C). In Supplementary Tables 2 and 3, the GSEA results' details are provided. All in all, we hypothesized that tumor immunology research should be focused on in efforts to pinpoint the root of the group with a high riskScore's bad prognosis.

### Comprehensive immune profiling of prognostic risk model based on CRs

#### Evaluating tumor immunogenicity and its role in prognostic risk models

**Fig. 4:**
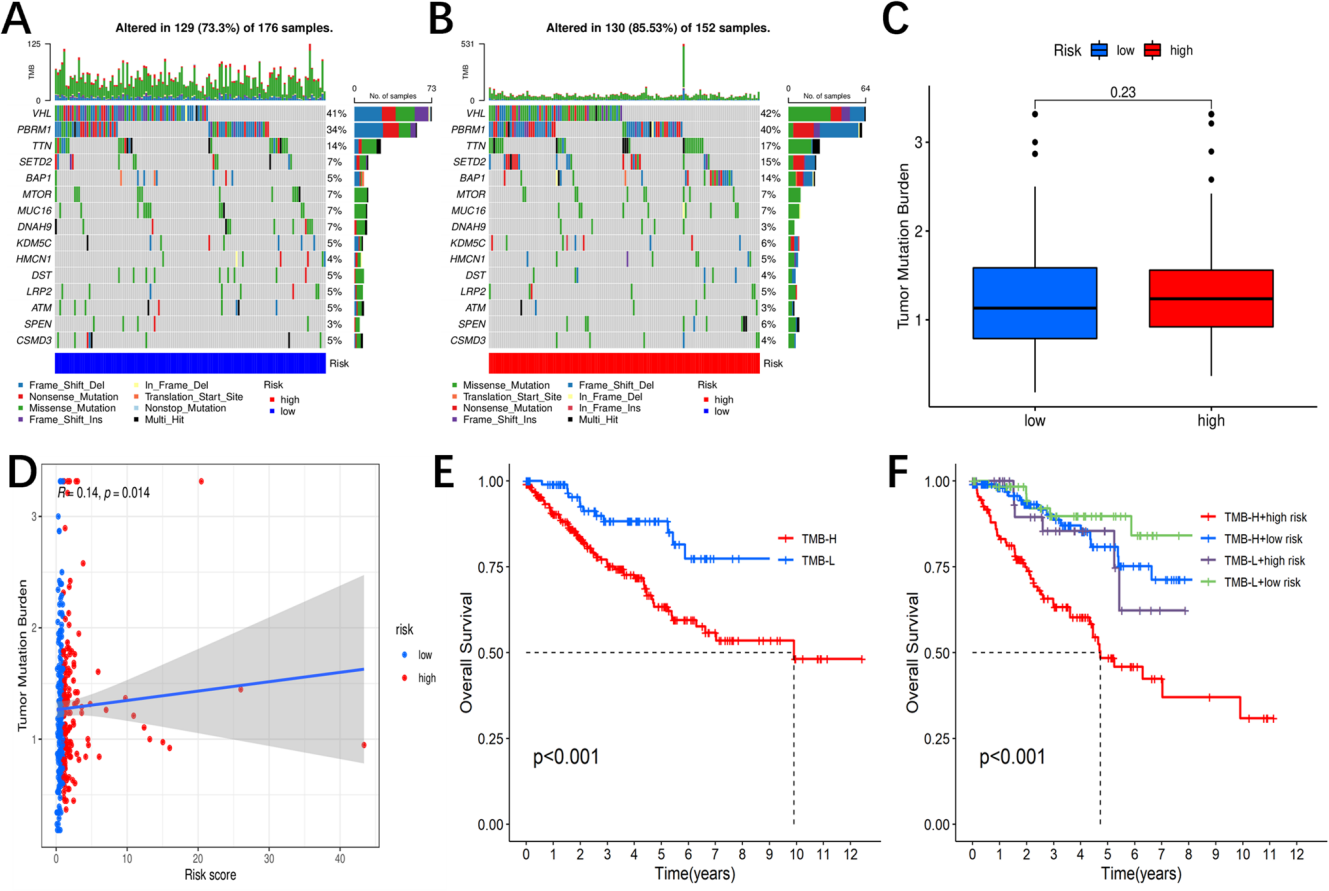
Immunogenicity analysis. **A**, **B** Waterfall plot shows the mutation distribution of the top 15 most frequently mutated genes in the group with high riskScore and low-risk group. **C** Difference in TMB between the high- and low-riskScore groups. **D** Correlation between the risk score and TMB. **E**, **F** Survival analysis of OS in different groups

Firstly, we tried to clarify whether tumor immunogenicity has differed and determine its role in the prognostic risk model. The rationale for using the Tumor Mutational Burden (TMB), an indirect measurement method, to measure immunogenicity was that a higher TMB was related to a higher neo-antigen load, and an increased neo-antigen load would result in higher immunogenicity [17, 18]. We used somatic mutation data to describe the enrichment of gene mutations in ccRCC and confirmed the different mutational pattern in these two groups. Figure 4A and B displayed the top 15 frequently changed genes. TMB abundance was greater in the group of ccRCC patients with high riskScore as compared to those with low riskScore (Fig. 4C). The correlation between the riskScore and TMB was shown to be positive (Fig. 4D). Patients with high TMB showed a significantly lower OS when ccRCC patients with low TMB and high TMB were compared (Fig. 4E). Additionally, high-TMB and high-riskScore individuals demonstrated much worse OS compared to those in the other groups (Fig. 4F).

#### Assessing differences in infiltrating immune cell levels between riskScore groups

**Fig. 5:**
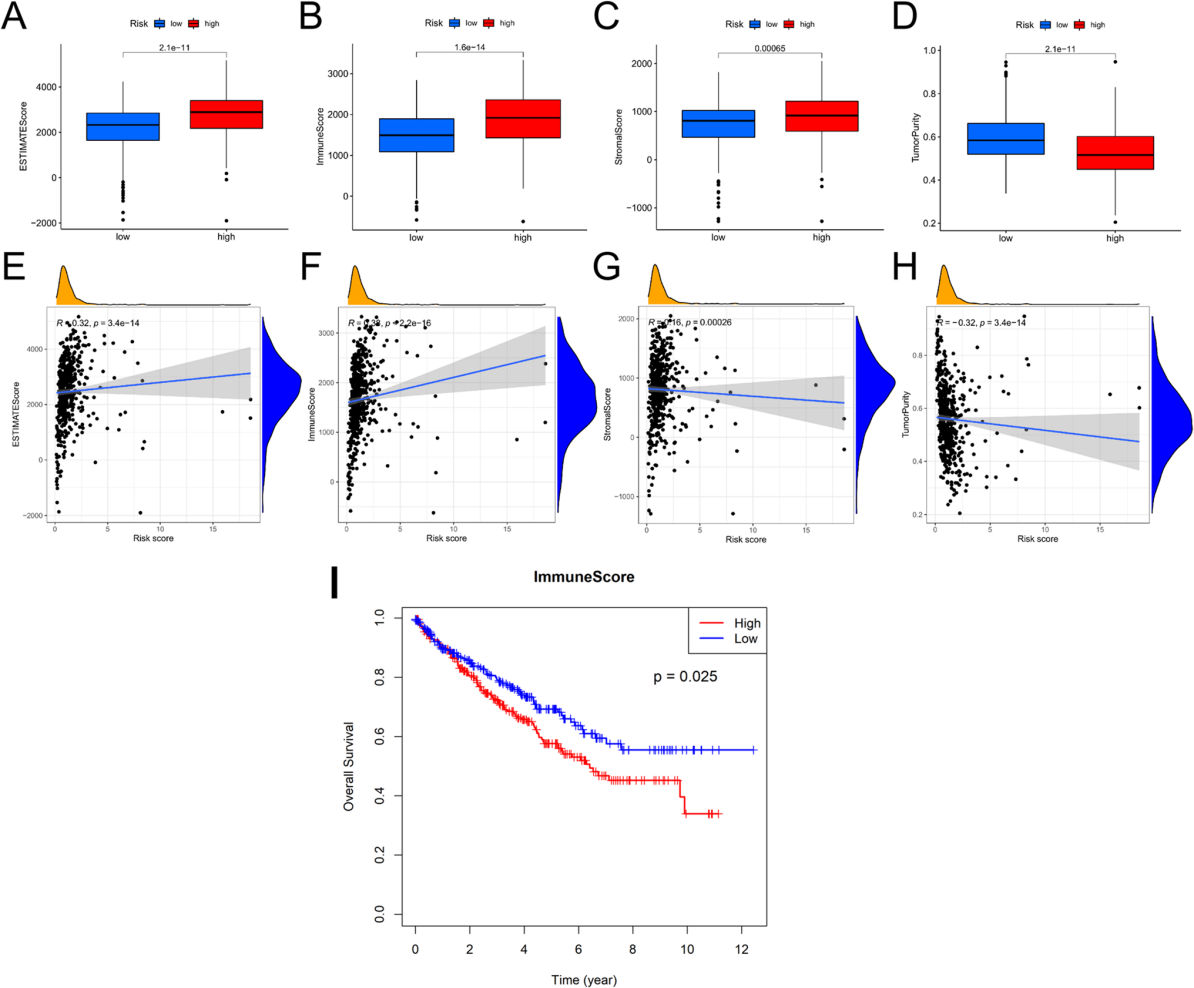
Analysis of the tumor immune microenvironment. **A**–**D** Box plots comparing ESTIMATEScore, ImmuneScore, StromalScore and TumorPurity between the low- and high-riskScore groups, respectively. **E**–**H** Correlation between riskScore and ESTIMATEScore, ImmuneScore, StromalScore, and TumorPurity, respectively. **I** Kaplan–Meier curves of overall survival between high and low ImmuneScore patients

Secondly, we thoroughly analyzed the model’s differences between the infiltrating immune cells’ levels of two riskScore groups. The immune response to malignancies is significantly impacted by the tumour microenvironment [19]. The TumorPurity, StromalScore, ImmuneScore, and ESTIMATEScore were thus produced by using the ESTIMATE algorithm. A higher ImmuneScore or StromalScore indicates a higher presence of immune or stromal cells within the TME, respectively. Combining these scores, the ESTIMATEScore reflects the overall proportion of immune and stromal cells. Our findings revealed that the high-riskScore group had lower TumorPurity and higher StromalScore, ImmuneScore, and ESTIMATEScore (P < 0.001) (Fig. 5A–D). I Specifically, ImmuneScore and ESTIMATEScore positively correlated with the riskScore, whereas StromalScore and TumorPurity were inversely related (Fig. 5E–H). Notably, only a high ImmuneScore was linked to a poor prognosis (Fig. 5I, Fig S6). We hypothesize that an elevated model risk score induces immune cell infiltration, thereby leading to a poor prognosis.

**Fig. 6:**
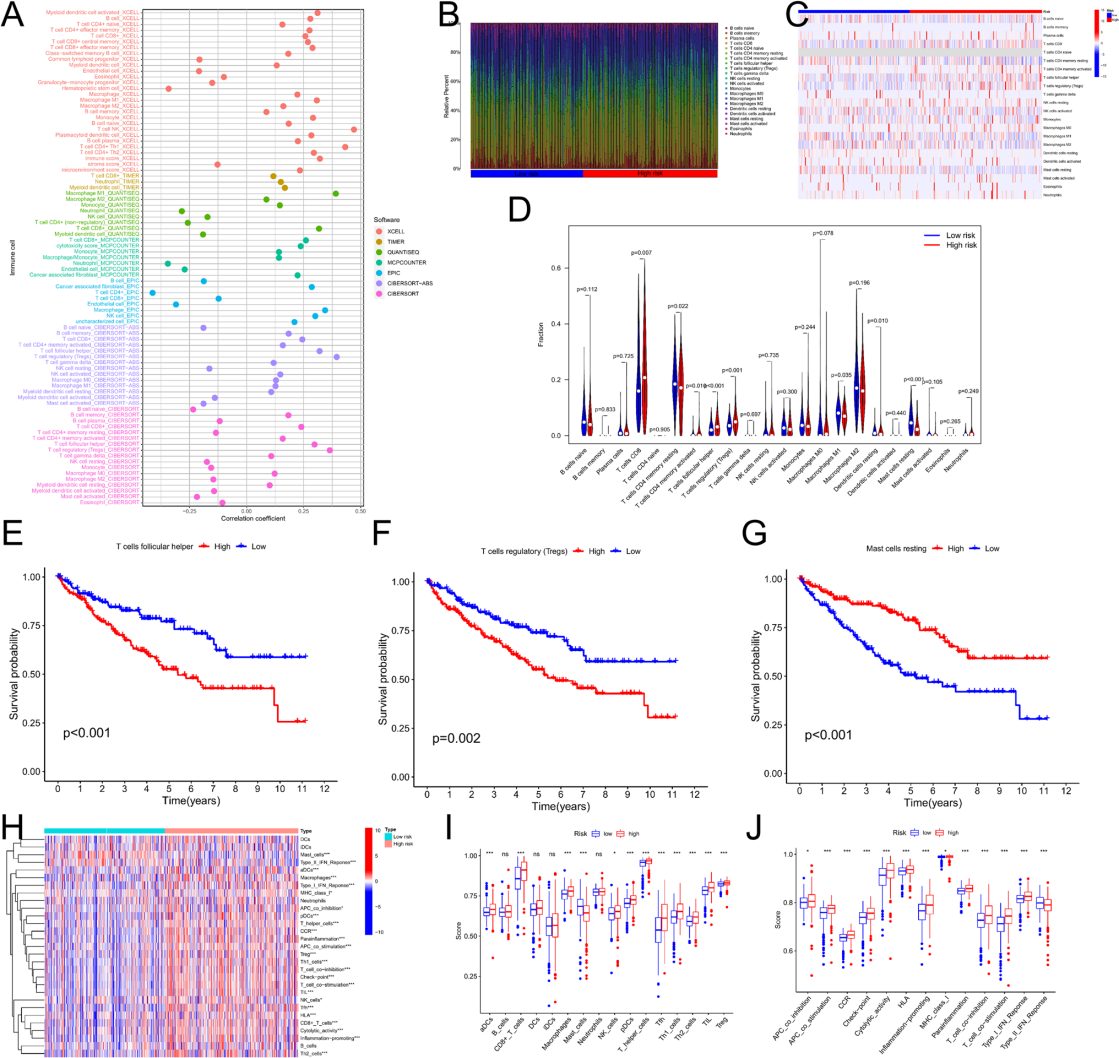
Analysis of the immune infiltration pattern. **A** Correlations between risk score and immune cell infiltrations by following software: XCELL; TIMER; QUANTISEQ; MCPCOUNTER; EPIC; CIBERSORT-ABS and CIBERSORT. **B** Bar graphs exhibiting the distribution of tumour-infiltrating immune cells between the high-riskScore and low-riskScore groups based on CIBERSORT algorithm. **C** Heat map of immune cell infiltration landscape in the high-/low-riskScore groups based on CIBERSORT algorithm. **D** Differences in tumour-infiltrating immune cells in the risk groups. **E**–**F** survival analysis show the prognosis of T cells follicular helper, T cells regulatory (Tregs) and Mast cells resting. **H** ssGSEA scores of immune cells and immune function in the risk group. **I**-**J** ssGSEA scores of immune cells and immune function in the risk group. *p < 0.05, **p < 0.01, and ***p < 0.001

To further analyze immune cell infiltration in ccRCC specimens, we utilized several algorithms: MCPCOUNTER, CIBERSORT-ABS, QUANTISEQ, XCELL, EPIC, TIMER, and CIBERSORT. The Pearson correlation test showed significant associations between the riskScore and various immune cell infiltrations (|R|≥ 3) (Fig. 6A). Specifically, we observed positive correlations between the riskScore and 12 types of tumor-infiltrating cells (TICs), such as Macrophage M1_QUANTISEQ, T cell NK_XCELL, and T cell CD8 + _QUANTISEQ, while four TICs, including T cell CD4 + _EPIC and Neutrophil_MCPCOUNTER, exhibited negative correlations (Supplementary Table 3).

The CIBERSORT algorithm confirmed the prognostic relevance of immune cell infiltration by constructing 21 immune cell profiles (Fig. 6B, C). We found that CD8 + T cells, regulatory T cells (Tregs), activated memory CD4 + T cells, and follicular helper T (Tfh) cells were highly expressed in the high-riskScore group, while resting CD4 memory T cells, resting mast cells, M1 macrophages, and resting dendritic cells were lowly expressed (Fig. 6D). Importantly, high levels of Tfh and Tregs correlated with poor prognosis, whereas high levels of resting mast cells indicated a favorable prognosis (Fig. 6E–G).

To support these findings, we conducted a single-sample Gene Set Enrichment Analysis (ssGSEA) for TICs (Fig. 6H). The results showed significant differences in the infiltration levels of 12 immune cell types between the high and low riskScore groups, excluding B cells, dendritic cells (DCs), immature DCs (iDCs), and neutrophils (p < 0.05) (Fig. 6I). In the high-riskScore group, except for mast cells, 11 immune cells, such as aDCs, NK cells, TILs, Tregs, pDCs, CD8 + T cells, Th cells, Th1, Th2, macrophages, and Tfh, were more abundantly infiltrated (p < 0.05) (Fig. 6I). Additionally, 13 immune-related pathways were more activated in the high-riskScore group, except for Type II IFN Response (p < 0.05) (Fig. 6J). These results indicate that higher levels of specific immune cells and pathways, including aDCs, T cell co-stimulation, APC co-inhibition, CCR, parainflammation, Tfh, Th1, Th2, Type I IFN Response, Treg, TIL, inflammation-promoting, T cell co-inhibition, and CD8 + T cells, along with lower levels of B cells, neutrophils, HLA, DCs, Type II IFN Response, pDCs, mast cells, iDCs, and NK cells, are associated with poorer prognosis in ccRCC patients (Fig. S7).

#### Investigating the impact of immune treatment on prognostic risk models

**Fig. 7:**
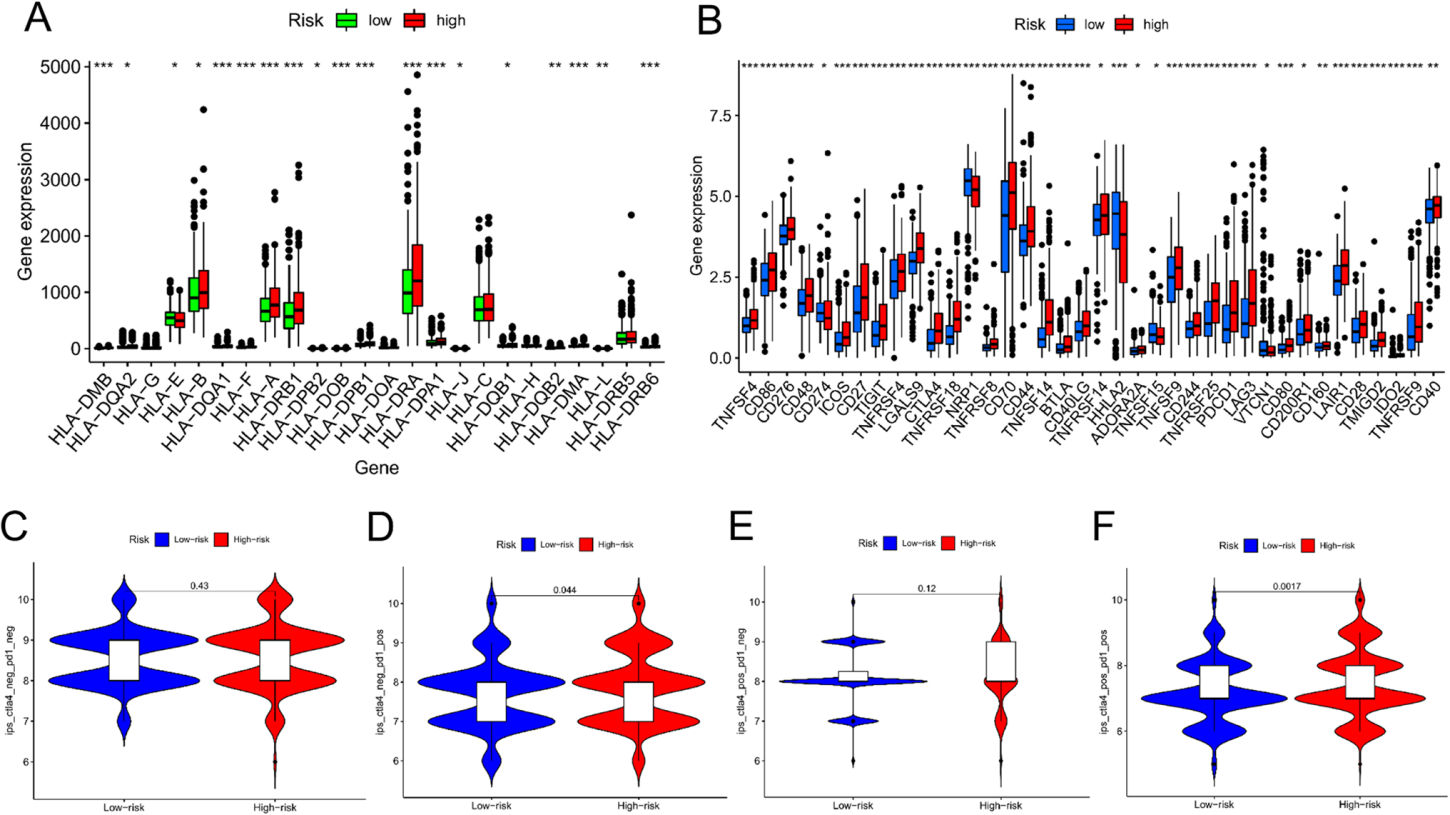
Immune therapy and Gene–drug sensitivity analysis. **A**, **B** Differences in expression of HLA-related genes and common immune checkpoints in the risk groups. **C**–**F** The relationship between risk group and immunophenoscore (IPS)

Finally, we investigated the relationship between immune treatment and the prognostic risk model. The responsiveness of kidney cancer to immunotherapies is well-recognized [20]. So, we evaluated connections between our model and several important factors, such as immune checkpoint molecules and Human Leukocyte Antigen (HLA). There were more checkpoint and HLA-related gene expression in the group with a high riskScore (Fig. 7A, B). Moreover, we investigated the association between riskScore and cancer immunotherapy response. To determine how effectively riskScore can forecast the curative impact of immune checkpoint blockade antibodies, we investigated how the prognostic risk model and immunophenoscore (IPS) related by employing immunophenogram analysis. Our results demonstrated that the group with high riskScore IPS was significantly higher in the anti‐PD1 and anti‐PD1 + CTLA4 (both P < 0.0001) (Fig. 7C–F). These findings suggest that PD1 monoclonal antibodies or an immune cocktail therapy (anti-PD-1 combined with anti-CTLA4) were more successful in treating individuals with a high riskScore. We next used an independent drug sensitivity test, which uses model genes to predict potentially sensitive drugs. According to the findings, risk CRs that had a high expression in the group with a high riskScore were positively correlated with drug sensitivity, including Nelarabine, Vorinostat, 6THIOGUANINE, Parthenolide, Hydroxyurea, Cladribine, Cytarabine, Thiotepa, Chlorambucil, and Triethylenemelamine (Fig. S8). Our findings may provide a cutting-edge pharmacological treatment plan for both high- and low-riskScore patients.

Collectively, according to our data, our model is likely to be an important determinant of tumor immunogenicity, infiltrated immune cells, and response to immunotherapies in ccRCC.

#### AURKB as a key prognostic marker and its role in immune modulation

Using log2| FC|> 1 and FDR < 0.05 as cutoff values, differently expressed CRs (DECRs) between low riskScore and high riskScore group were found. We screened 6 DECRs, including 2 low-risk genes (log2 FC < 1, high expression in low-riskScore group) and 4 high-risk genes (log2 FC > 1, high expression in high-riskScore group) (Supplementary Table 4). Interestingly, AURKB, one of the high-risk genes, was also one of the genes included in our prognostic model. Consequently, we selected AURKB as a key CR for further analysis.

**Fig. 8:**
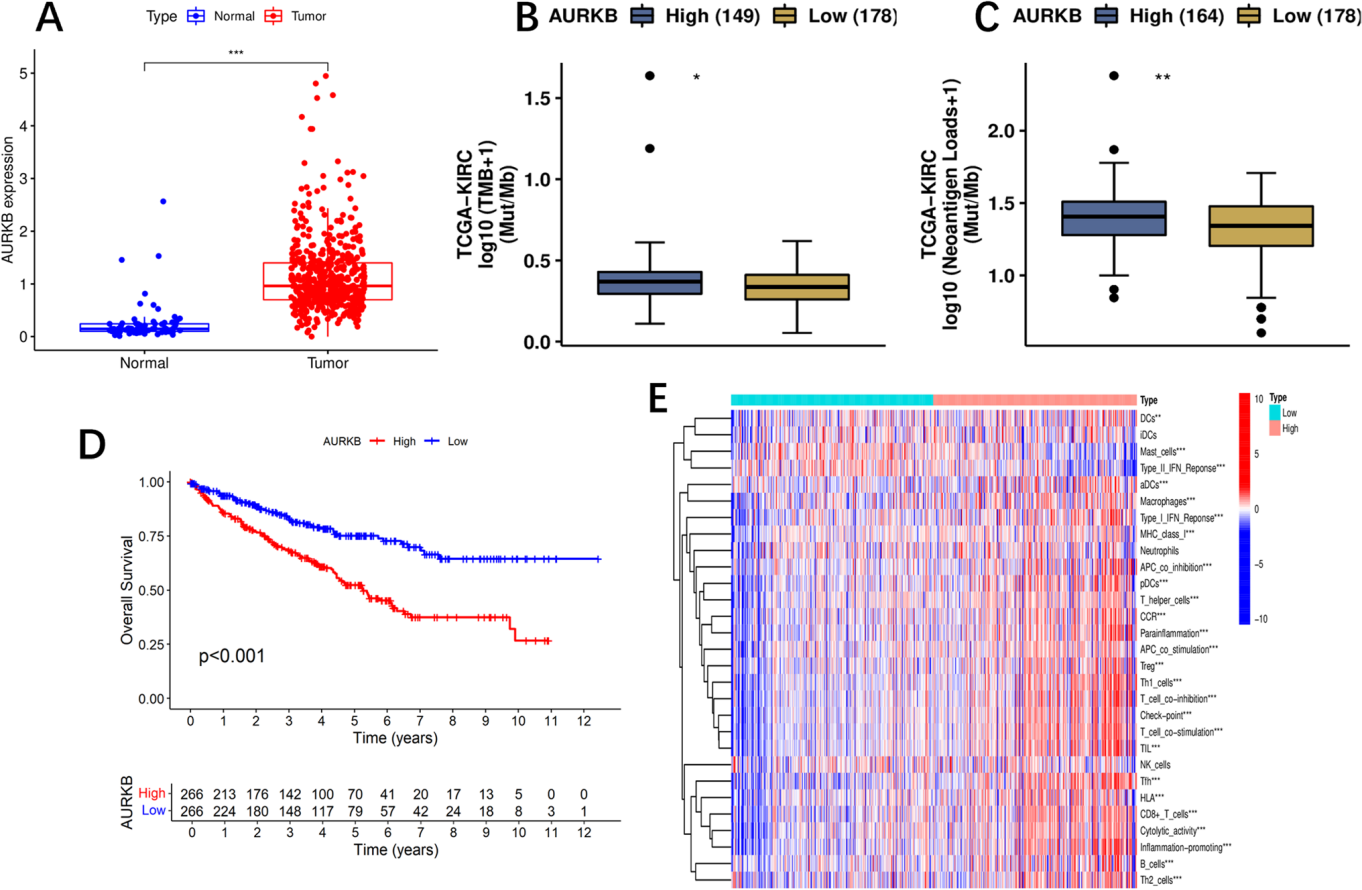
The role of AURKB. **A** the expression of AURKB in ccRCC and normal tissues (tumor in red and normal in blue). **B**, **C** TMB and Neoantigen Loads analysis of AURKB based on CAMOIP. **D** survival analysis show the prognosis of AURKB. **E** ssGSEA scores of immune cells and immune function in expression of AURKB

AURKB was highly expressed in tumors and associated with poor prognosis (Fig. 8A, D). Further investigation revealed that AURKB plays a significant role in immunogenicity and immune cell infiltration (Fig. 8B–E). Specifically, high AURKB expression correlated with increased infiltration levels of various immune cell types, including CD8 + T cells and regulatory T cells (Tregs) (Fig. 6E). These findings suggest that AURKB may influence tumor progression and patient prognosis by modulating the tumor immune microenvironment.

In summary, our analysis not only confirmed the pivotal role of AURKB in our prognostic model but also highlighted its importance in immunogenicity and immune cell infiltration. This positions AURKB as a crucial target for studying tumor immune evasion mechanisms and developing novel immunotherapeutic strategies.

## Discussion

ccRCC is a highly aggressive kidney malignancy, with one-third of patients presenting metastases at diagnosis [21]. Despite recent therapeutic advancements, relapse and mortality rates remain high, particularly for individuals with advanced or metastatic conditions, who face a grim prognosis [9]. Numerous studies have highlighted the diverse roles of CRs in the progression of ccRCC [22–26]. Therefore, it is crucial to develop CR-specific biomarkers for therapy and prognosis monitoring in ccRCC patients.

In this study, we identified 65 CRs with distinct expression patterns in ccRCC tumors compared to benign tissues using data from the TCGA database. Following that, two subclasses were produced using non-negative matrix factorization clustering based on these 65 CRs. Next, 20 CRs associated with progression in ccRCC between the two subclasses were identified. We used these CRs to develop a novel prognostic model for ccRCC, dividing 521 KIRC samples into two subgroups. By applying the LASSO regression algorithm, we created a prognostic profile based on five CRs. Our model’s predictive power was demonstrated through PCA, survival, and ROC analyses, and it was directly compared with other clinical prognostic models, showing superior predictive accuracy. Univariate and multivariate Cox analyses confirmed that our model is an independent predictive factor. The correlation between clinicopathological characteristics and riskScore, stratified survival analyses, and validation using the ICGC dataset all demonstrated the model’s broad applicability.

To further understand the mechanisms underlying this signature's role in ccRCC, we performed GO and GSEA analyses. These analyses revealed that several immune-related mechanisms were highly ranked in the high-riskScore group. The following sections detail our findings:

*Immunogenicity* Cancer is also considered a genetic disorder [27], with gene mutations directly impacting tumor progression and metastasis [28]. Tumor mutational burden (TMB), a recent immunotherapy biomarker, is one marker of tumor antigenicity [29]. Additionally, our research revealed that the landscape of somatic cell copy number alternations (SCNAs) varied in the different riskScore groups. TMB was more substantial in the group with a high riskScore compared to the low riskScore group, indicating that they had higher tumor immunogenicity, which aggravated high-TMB’s poor prognosis.

*Immune Infiltration* TME, which includes immune cells, plays a crucial role in cancer development and progression [30, 31]. Our data analysis revealed a distinct difference in the TME between high and low-riskScore groups. The high-riskScore group had a higher ImmuneScore and lower TumorPurity. Our findings agreed with those made by Zeng et al., who suggested that individuals with lower immune scores tend to have longer life expectancies than patients with higher immune scores [32], and by Zhang et al., who suggested that aggressive behavior and poor prognosis were due to low tumor purity [33]. As an immunogenic malignancy, ccRCC promotes the penetration of immunosuppressive cells into the TME, mediating immune dysfunction [34]. The research of CRs about the TME in ccRCC is still incomplete. In this investigation, the model-based riskScore and the infiltrating immune cells were shown to be positively correlated. This result supports a prior study's conclusion that individuals with high-risk gliomas who have more immune cell infiltration have a worse prognosis [35]. The high-riskScore group showed higher immune cell infiltration, which is linked to a worse outcome.

*Immune therapy* The success of immunotherapy relies on the ability of HLAs to present tumor neoantigens on the cell surface [36]. The field of immuno-oncology has undergone a revolution thanks to immune-checkpoint-based cancer immunotherapy [37]. Our model’s assessment of HLA-related gene expression and immune checkpoints revealed higher expression levels in the high-riskScore group. Blocking immune checkpoint molecules is a promising strategy for cancer therapy [38]. Prostate cancer, lung cancer, and ccRCC have all been studied using monoclonal antibodies that target different immune checkpoint inhibitors (such as CTLA4, PDCD1, and LAG3) [39]. However, immune checkpoint blockade (ICB) treatments are ineffective for a large number of advanced cRCC patients [40]. Importantly, our findings suggest that high-riskScore patients respond better to PD-1 inhibitors, CTLA4 inhibitors, or a combination of both. Drug sensitivity analysis indicated that specific chemotherapy drugs are more effective in high-riskScore patients, offering new insights into treatment combinations.

Our findings suggest that the prognostic model has significant potential for clinical use, predicting prognosis, immunogenicity, TICs, immune treatment, and chemosensitivity. Moreover, our study indicates that immune infiltration caused by elevated immunogenicity contributes to a poor prognosis in the high-riskScore group.

To gain insight into the molecular mechanisms, we focused on AURKB, a key mRNA that was highly expressed in the high-riskScore group and part of our prognostic model. AURKB, a component of the chromosomal passenger complex, plays a role in cell mitosis [41], in addition to the spindle assembly checkpoint, cytokinesis, chromosomal condensation, chromosome-microtubule interaction, and sister chromatid cohesion [42]. Additionally, it has been demonstrated that AURKB is crucial for tumor development [43], progression [44], and chemotherapy response [45]. AURKB is a famous molecular target, and clinical trials are investigating selective inhibitors [46, 47]. Currently, research is being done on AURKB as a potential therapeutic target for several tumour types, including leukemia, prostate cancer (PC), gastric cancer (GC), breast cancer, and non-small cell lung cancer (NSCLC) [48–52]. AURKB can boost the development of ccRCC by activating a number of signaling pathways, including cell adhesion molecules (CAMs), natural killer cell-mediated cytotoxicity, the cell cycle, the intestinal immune network for IgA production, and cytokine-cytokine receptor interaction [53]. In ccRCC tissues, AURKB expression was increased [54]. A worse outcome was linked to high AURKB expression in cRCC patients, according to Li et al.'s research on the topic. Li et al. additionally found that ccRCC cell proliferation was suppressed by siRNA or inhibitors that blocked AURKB [55, 56]. However, basic research experiments have not addressed the possible function and mechanism of AURKB, and uncertainty exists over AURKB's position in ccRCC. We found that AURKB is related to immunogenicity and immune infiltration in the current study. It can be developed in ccRCC as a biomarker and a prognostic predictor.

Our study has several limitations. First, the data used for analysis came from public databases and were not independently validated. Second, experimental validation through in vitro and in vivo studies is necessary to confirm the functional role of AURKB in tumor progression and immune modulation. Finally, a multi-center clinical cohort is needed to demonstrate the prognostic model's reliability in practice.

Unlike other clinicopathologic signatures, our work developed a reliable prognostic prediction model based on just five CRs. This model effectively predicts overall survival, immunogenicity, immune infiltration, and responsiveness to immunotherapy and anticancer drugs in ccRCC patients. Our finding that AURKB promotes immunogenicity and immune infiltration opens new avenues for treatment strategies and enhanced antitumor immunity in ccRCC. This research also advances the development of biomarkers for prognosis prediction and treatment guidance in ccRCC.

### Supplementary Information

Supplementary File 1

Supplementary File 2

Supplementary File 3

Supplementary File 4

Supplementary File 5

Supplementary File 6

Supplementary File 7

Supplementary File 8

Supplementary File 9

Supplementary File 10

## Data Availability

The datasets used in this study's analysis are accessible in the TCGA project (https://tcga-data.nci.nih.gov/tcga/), UCSC Xena (http://xena.ucsc.edu/), ICGC(https://dcc.icgc.org/releases/current/Projects/KIRC-US), CIBERSORT web portal (http://CIBERSORT.stanford.edu/), The Cancer Immunome Atlas (https://tcia.at/home), the CellMiner database (https://discover.nci.nih.gov/cellminer/), the starBase database (http://starbase.sysu.edu.cn/index.php). Data analyzing of TMB and Neoantigen Loads can be found at Comprehensive Analysis on Multi-Omics of Immunotherapy in Pan-cancer (CAMOIP) (https://www.camoip.net).
