# Supplementary figures and images for: AURKB promotes immunogenicity and immune infiltration in clear cell renal cell carcinoma
Source: Discov Oncol. 2024 Jul 16;15:286. doi: 10.1007/s12672-024-01141-7 (PMC11252114; doi:10.1007/s12672-024-01141-7)

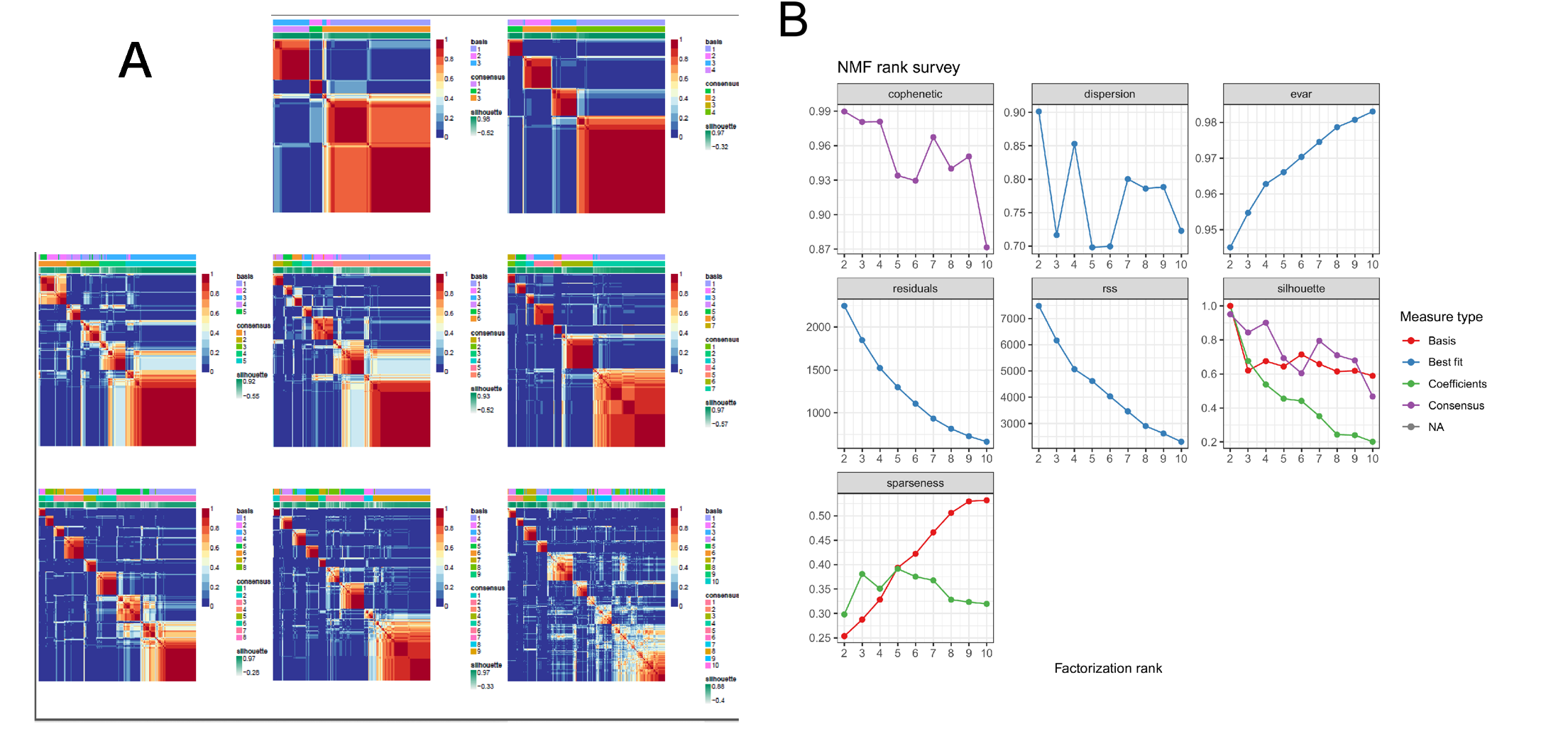

Supplement: Supplementary file 1 [file 12672_2024_1141_MOESM1_ESM.tif]

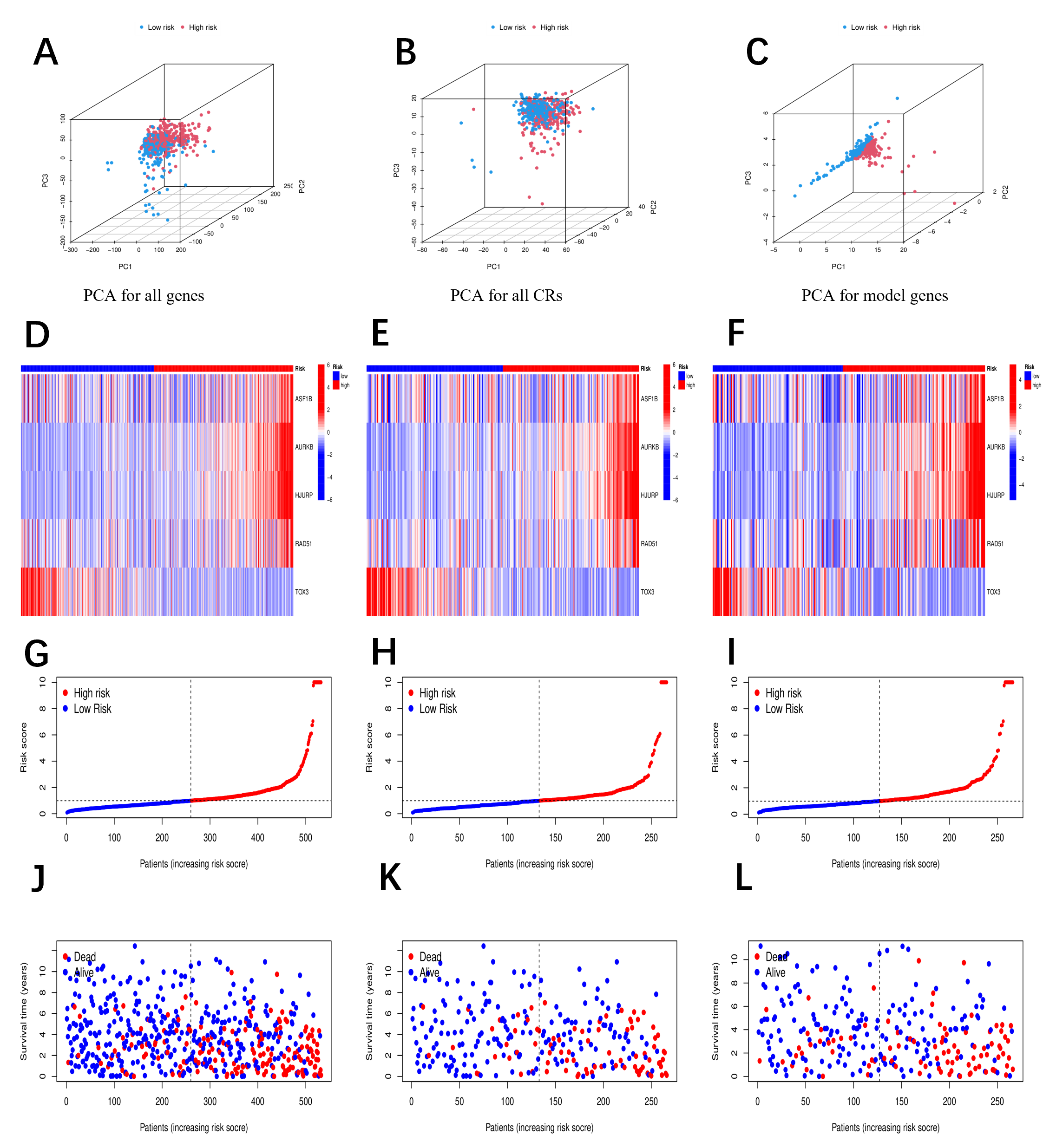

Supplement: Supplementary file 2 [file 12672_2024_1141_MOESM2_ESM.tif]

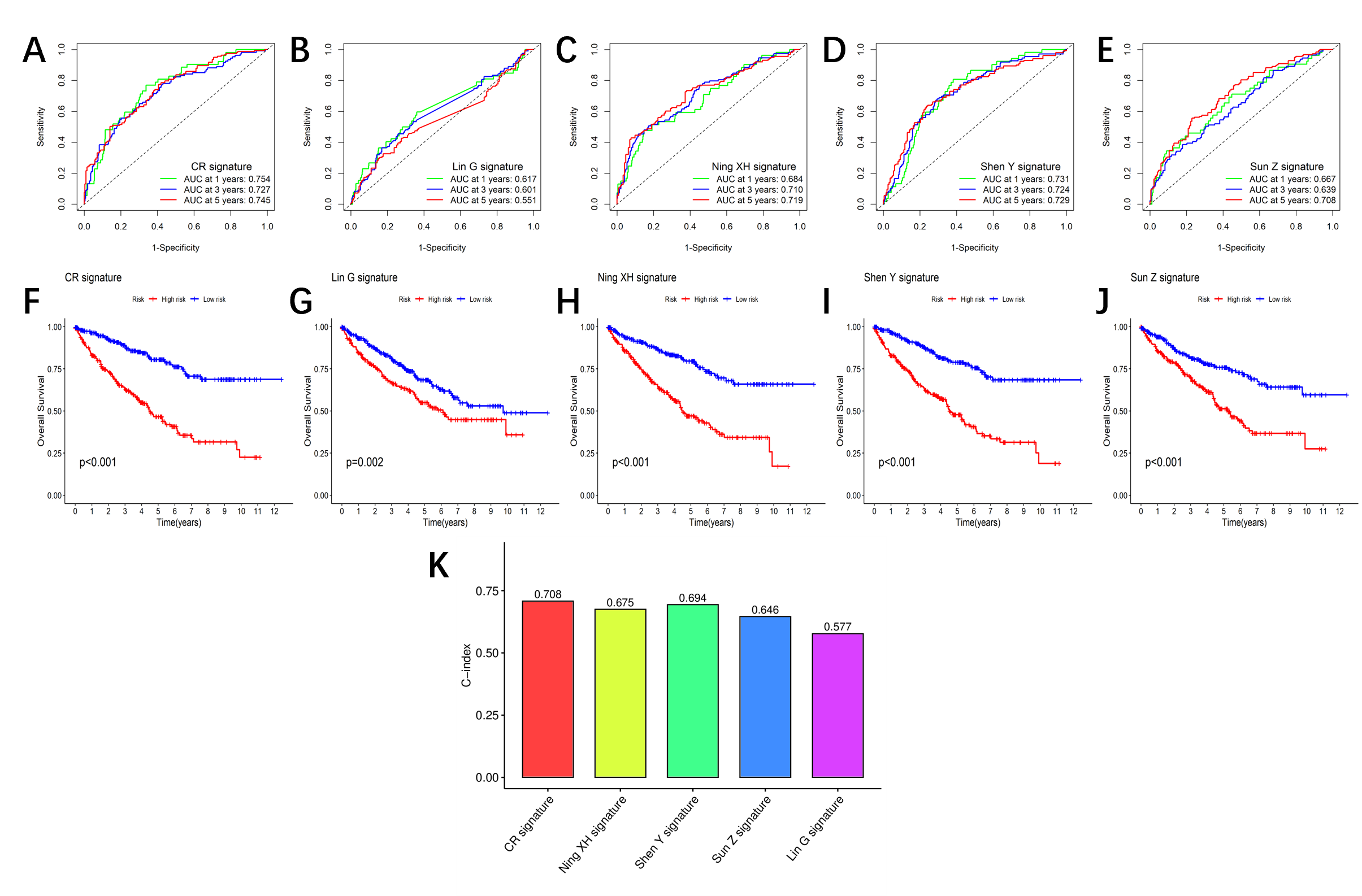

Supplement: Supplementary file 3 [file 12672_2024_1141_MOESM3_ESM.tif]

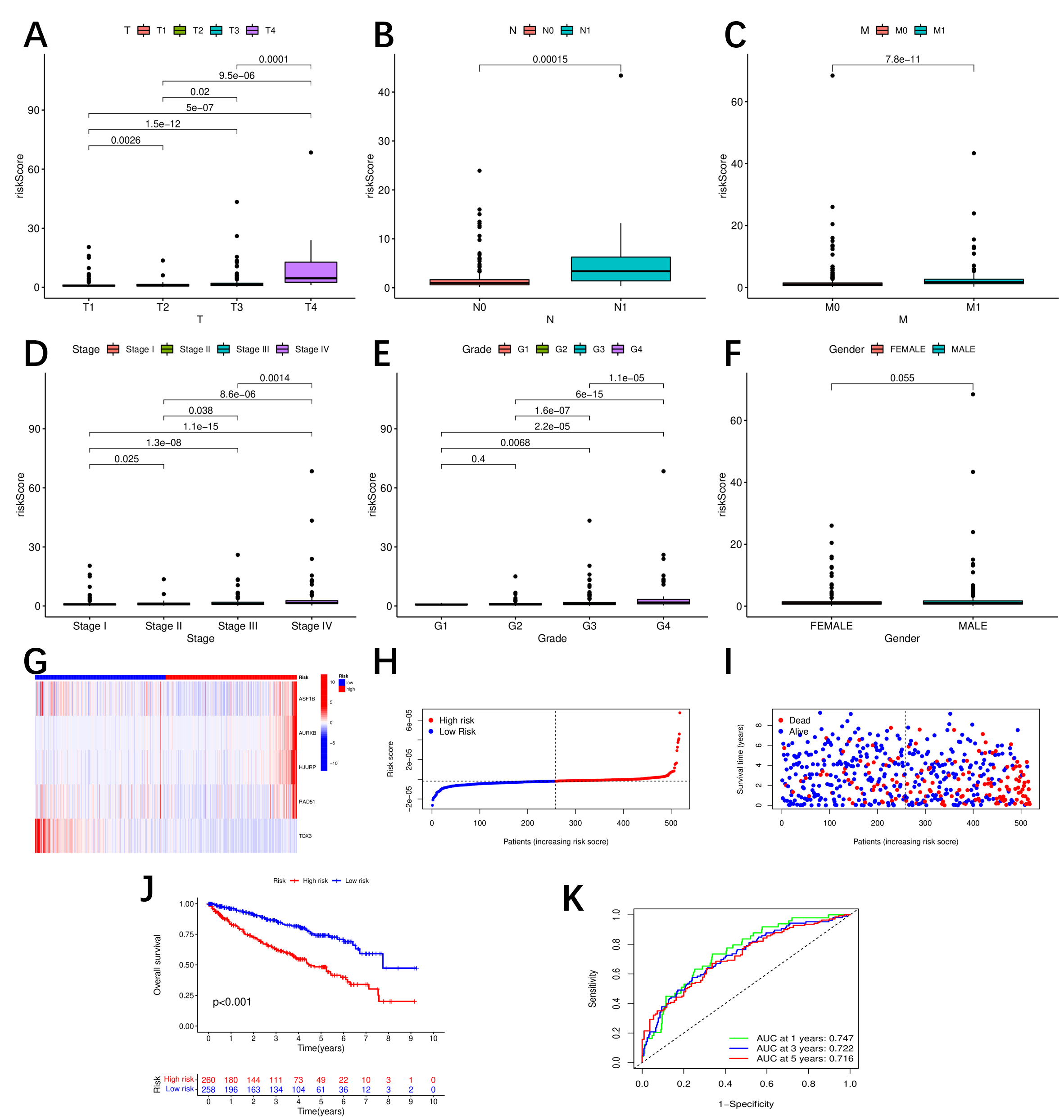

Supplement: Supplementary file 4 [file 12672_2024_1141_MOESM4_ESM.tif]

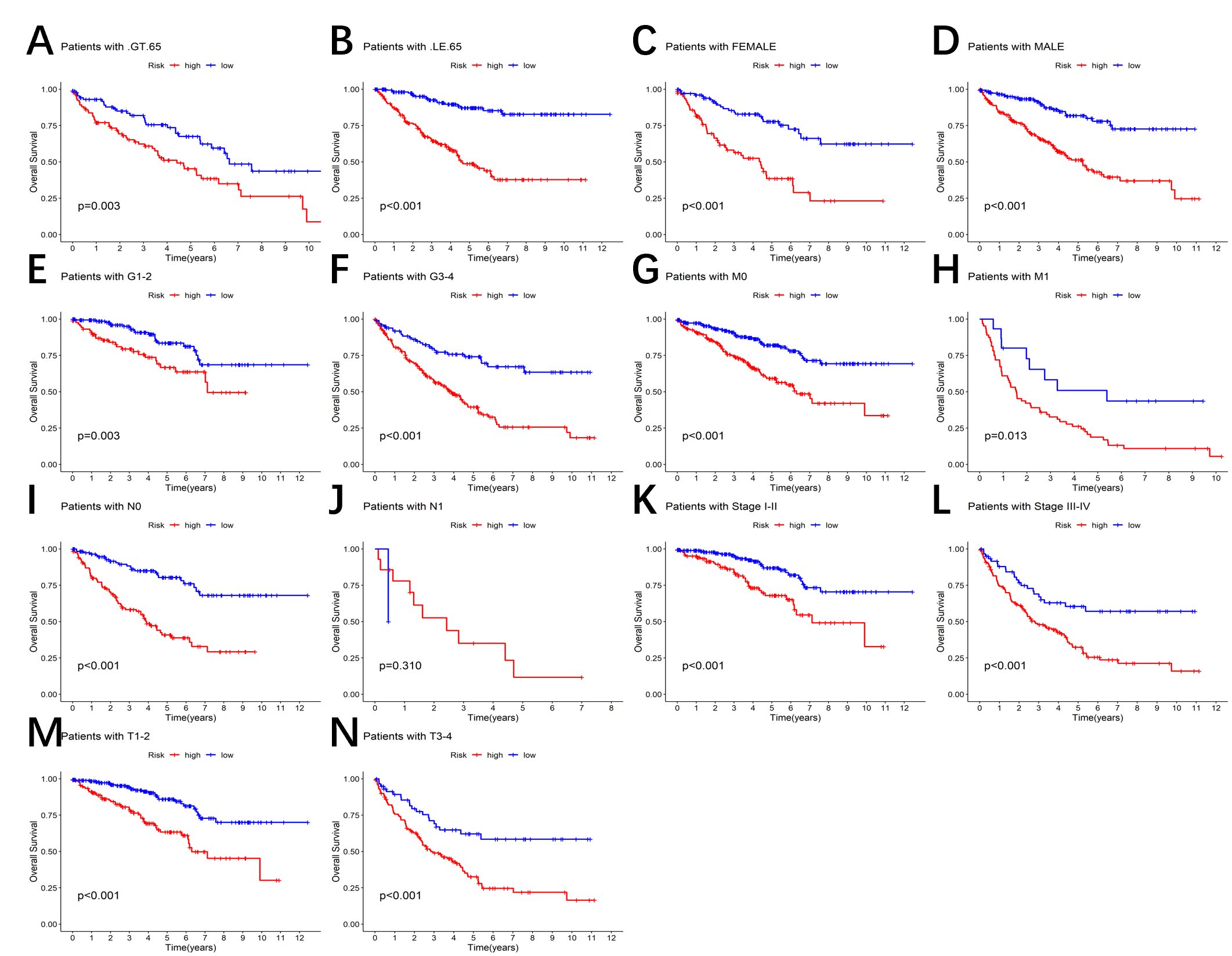

Supplement: Supplementary file 5 [file 12672_2024_1141_MOESM5_ESM.tif]

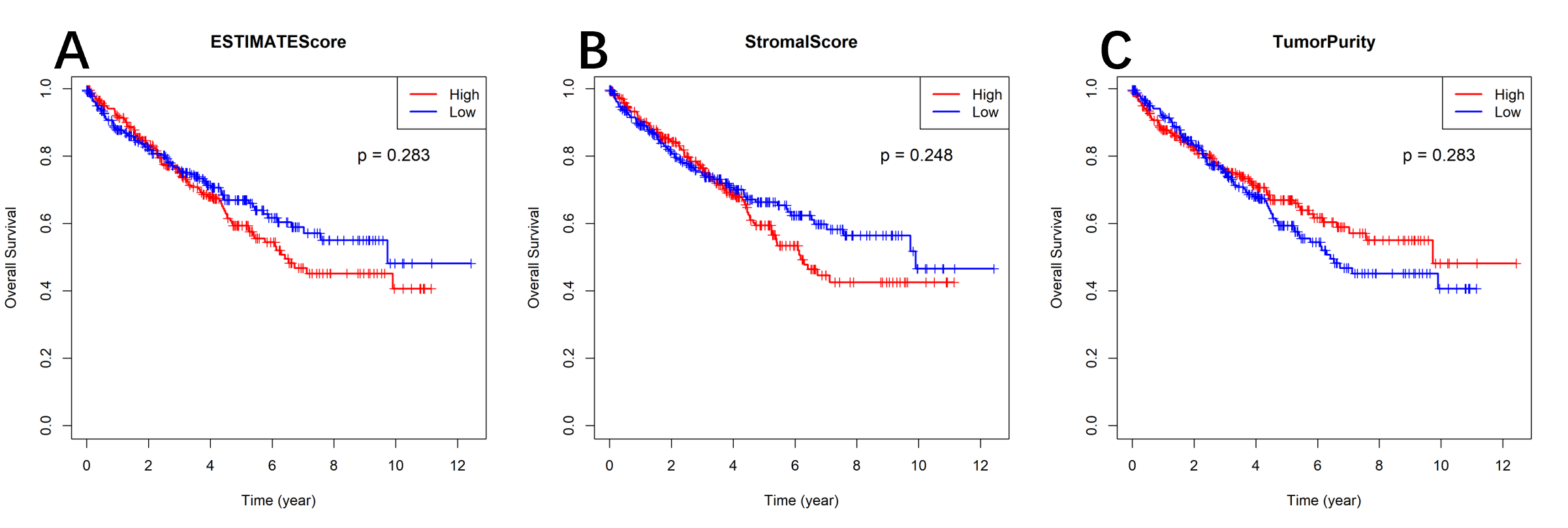

Supplement: Supplementary file 6 [file 12672_2024_1141_MOESM6_ESM.tif]

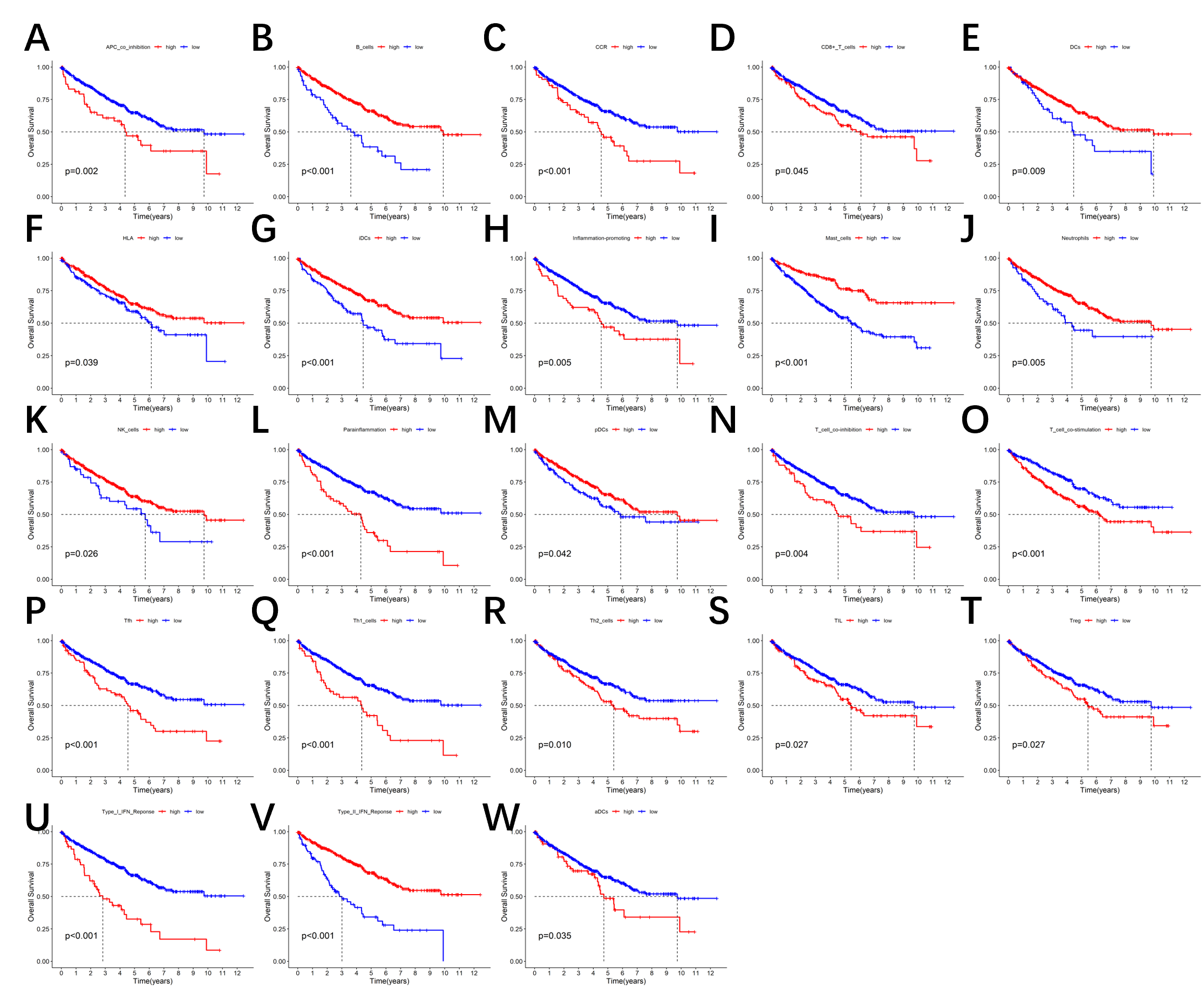

Supplement: Supplementary file 7 [file 12672_2024_1141_MOESM7_ESM.tif]

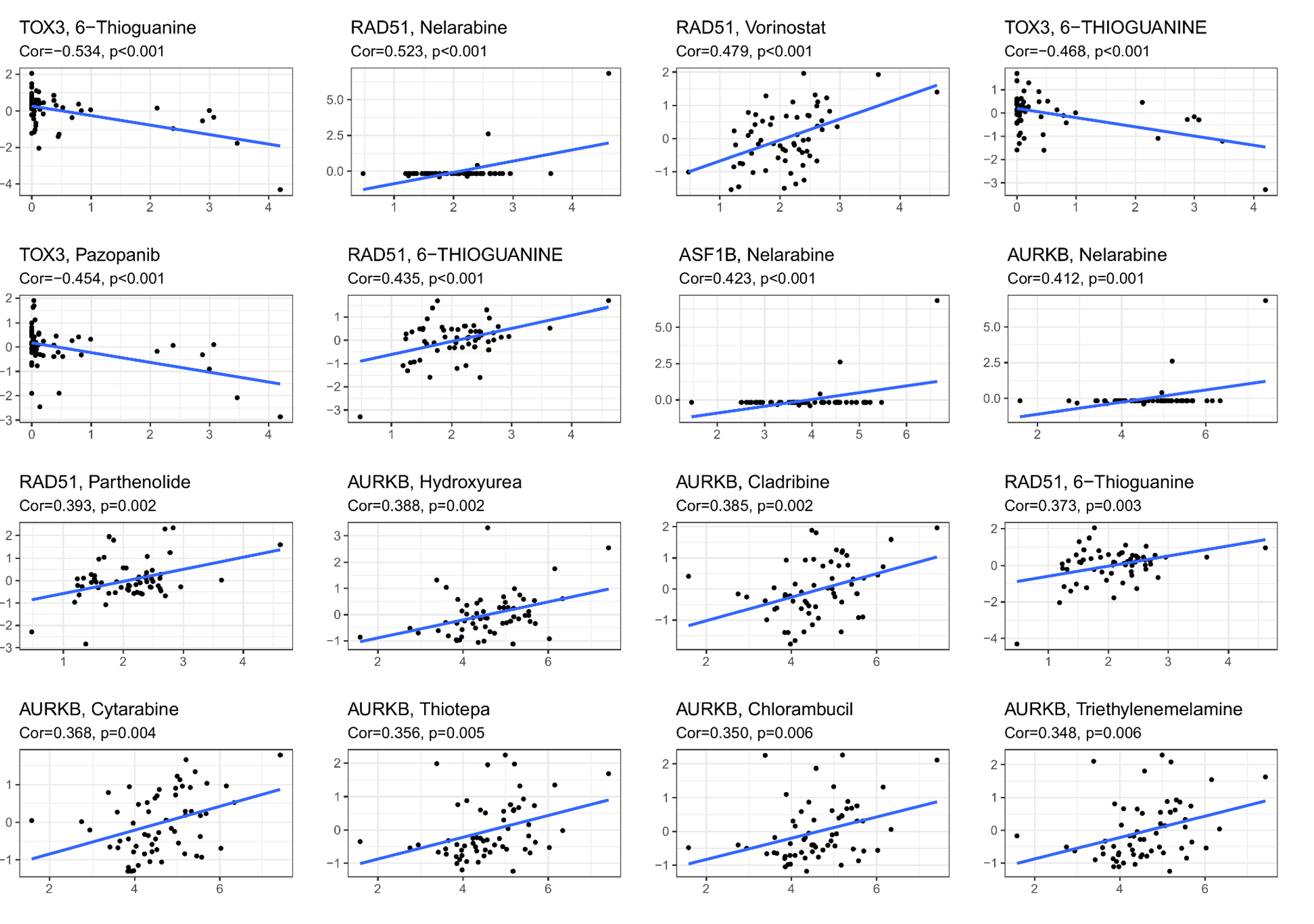

Supplement: Supplementary file 8 [file 12672_2024_1141_MOESM8_ESM.tif]
