# Supplementary material for: AURKB promotes immunogenicity and immune infiltration in clear cell renal cell carcinoma
Source: Discov Oncol. 2024 Jul 16;15:286. doi: 10.1007/s12672-024-01141-7 (PMC11252114; doi:10.1007/s12672-024-01141-7)
Supplement: Supplementary file 9 [file 12672_2024_1141_MOESM9_ESM.pdf]

Supplementary Materials

Fig S1. Identification ccRCC in TCGA cohort by consensus clustering. (A)k = 2-10 as the optimal number of clusters. (B) The relationship between cophenetic, dispersion and silhouette coefficients with respect to number of clusters.

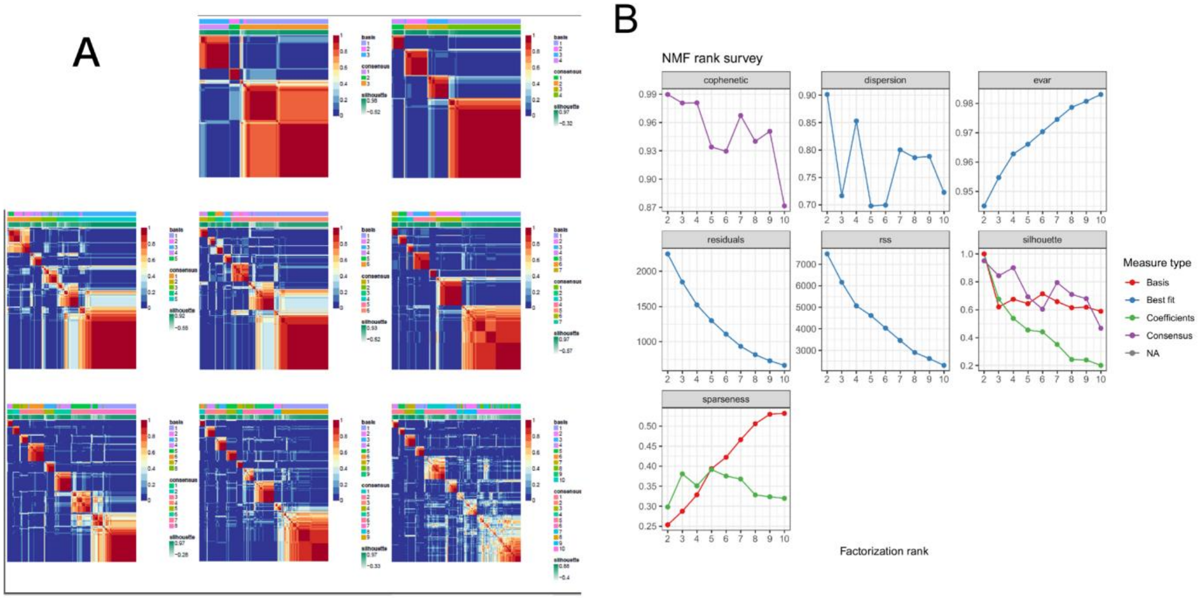

Fig S2. Evaluation the prognostic model. (A-C) The 3D scatterplot of the PCA showing the distribution of ccRCC patients based on all genes, all CRs and model genes. (D–F) Heatmap of the expression of 5 CRs in the entire, training, and testing sets, respectively. (G–I) Risk model of the entire, training, and testing sets, respectively. (J–L) Survival time and survival status in the entire, training, and testing sets, respectively.

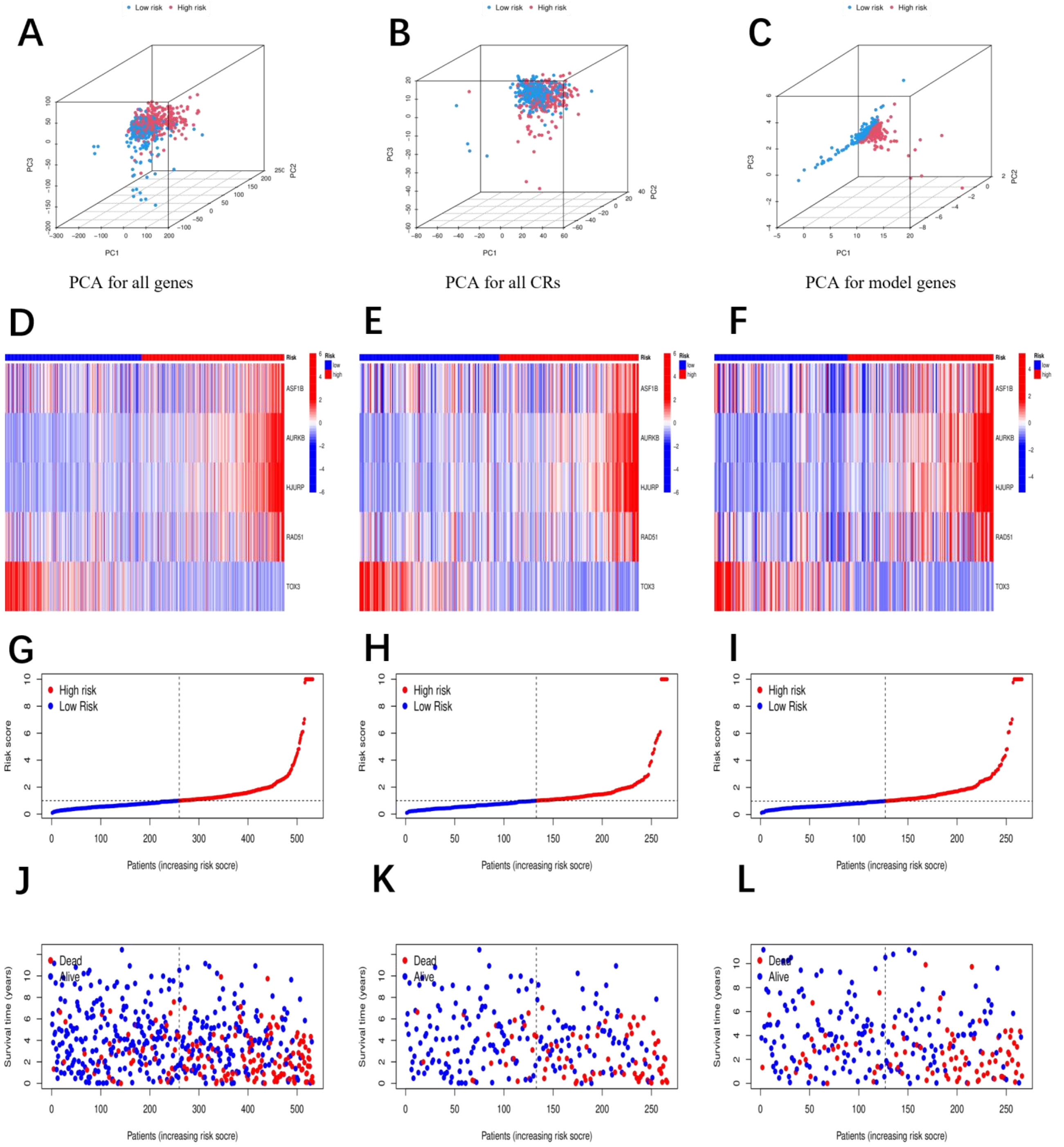

Fig S3. Comparison between different models. (A-E) 1-, 3-, and 5-year ROC curves of our model and other prognostic models. (F–J) Kaplan–Meier survival curves of patients with OS of our model and other prognostic models. (D) the Harrell's c-index for our model and other prognostic models.

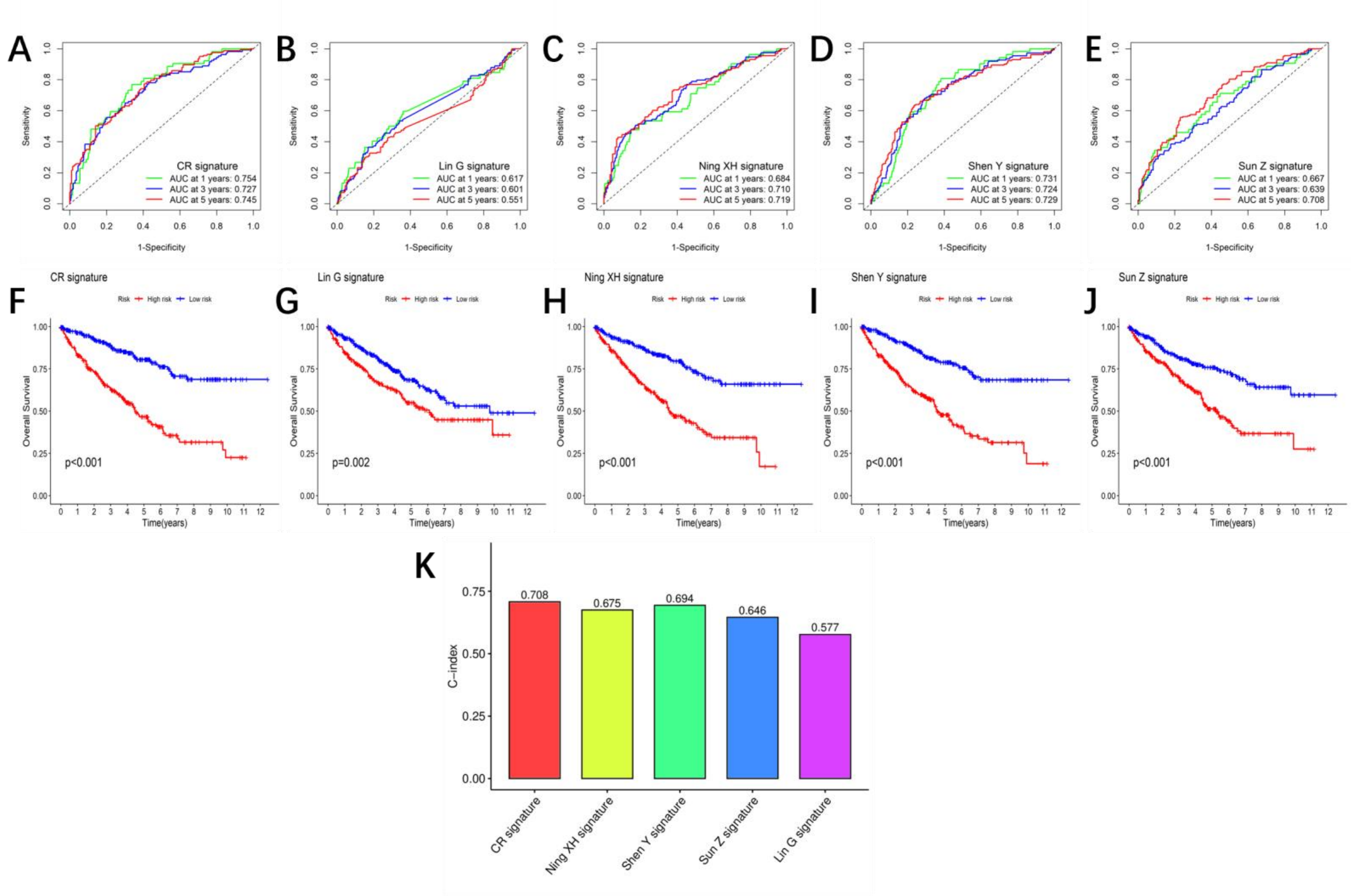

Fig S4. Clinical relevance and External Validation of prognostic model. (A-F) Correlation between signature and clinical characteristics. (G-K) Validation of ICGC cohort.

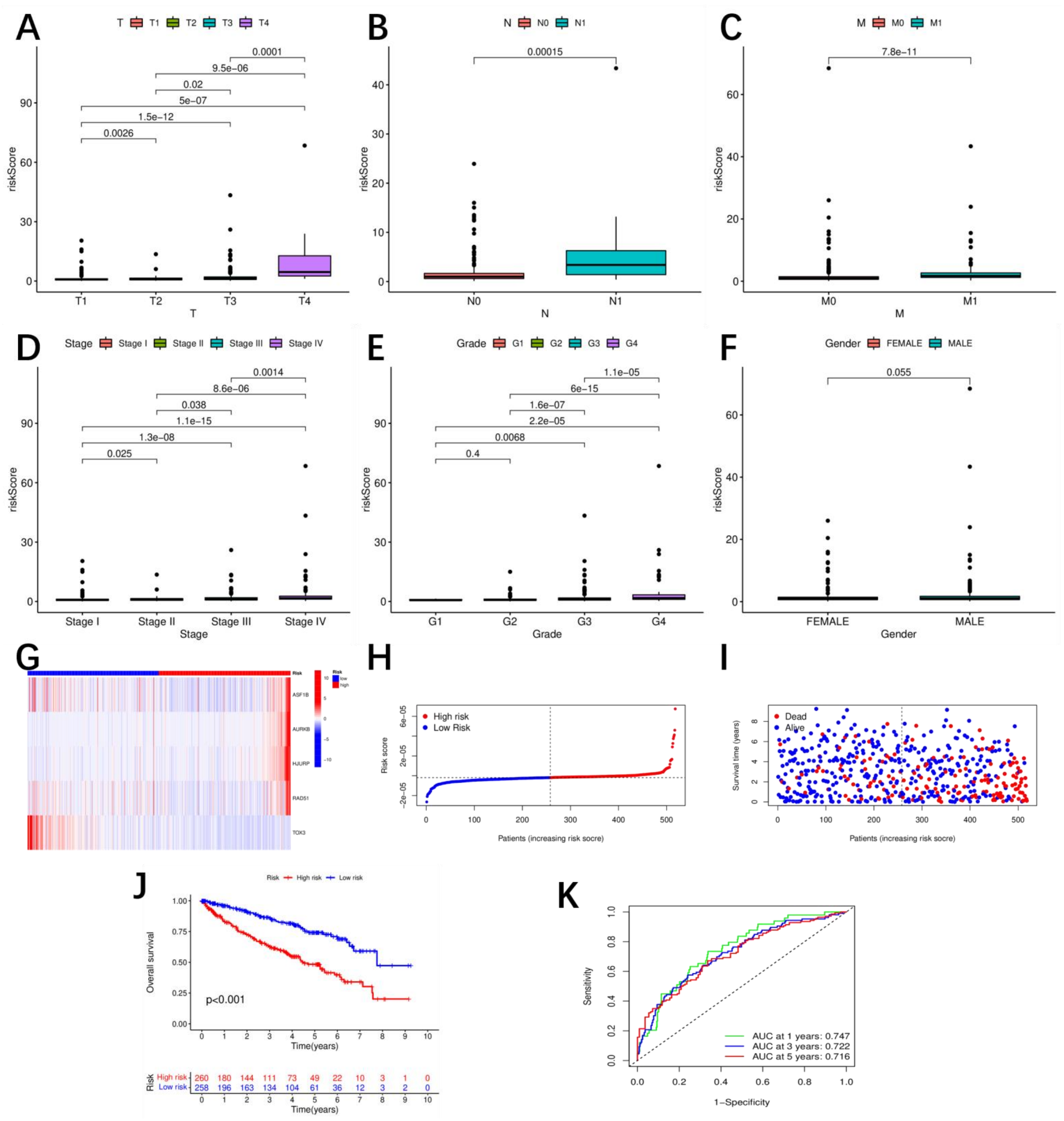

Fig S5. Survival stratification analysis of model in ccRCC: (A) age≤ 65; (B) age> 65; (C) female; (D) male; (E) G1−G2; (F) G3-G4; (G) M0; (H) M1; (I) N0; (J) N1; (K) stages I−II; (L) stages III−IV; (M) T1-T2; (N) T3-T4;

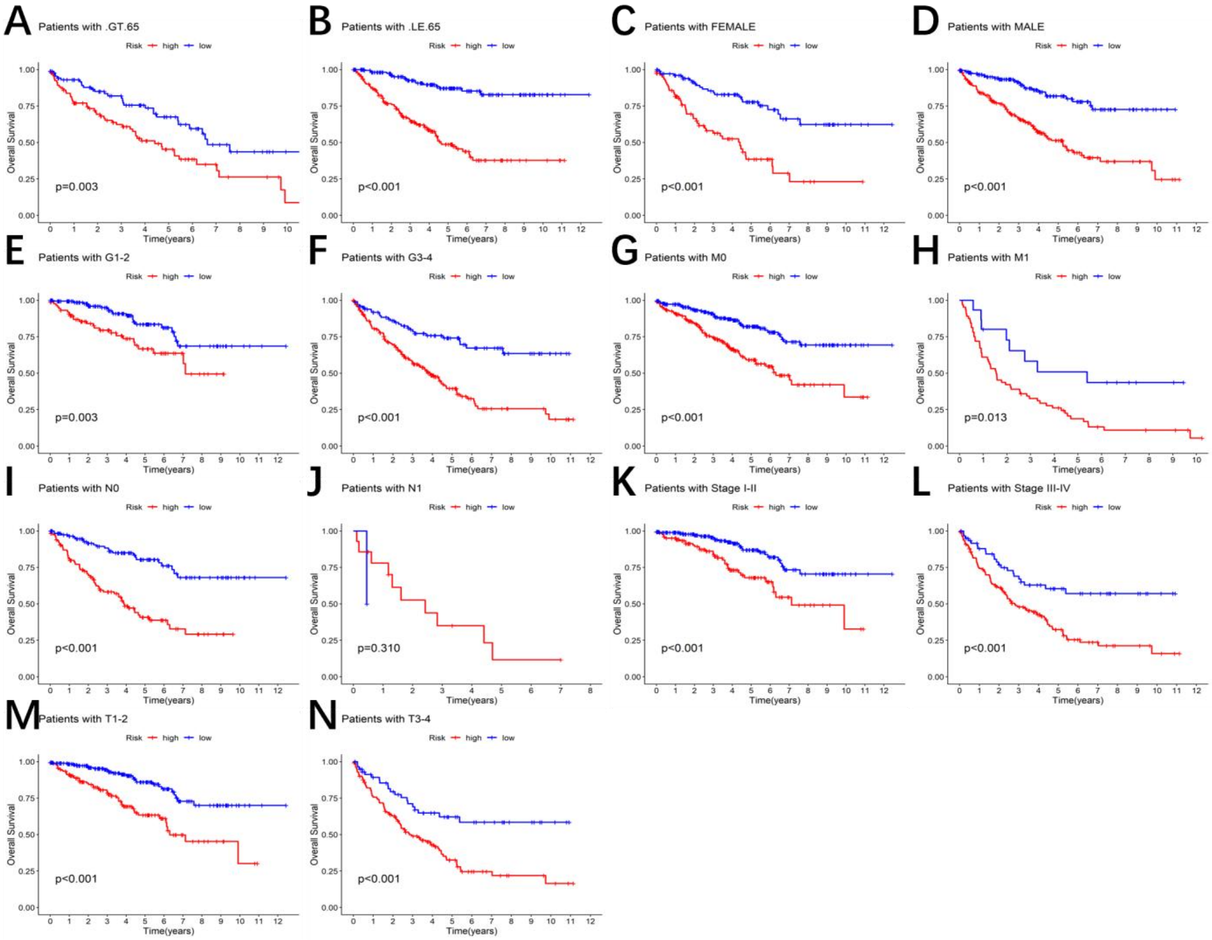

Fig S6. Kaplan–Meier curves of overall survival of TME. (A-C) Kaplan–Meier curves of ESTIMATEScore, StromalScore, TumorPurity, respectively.

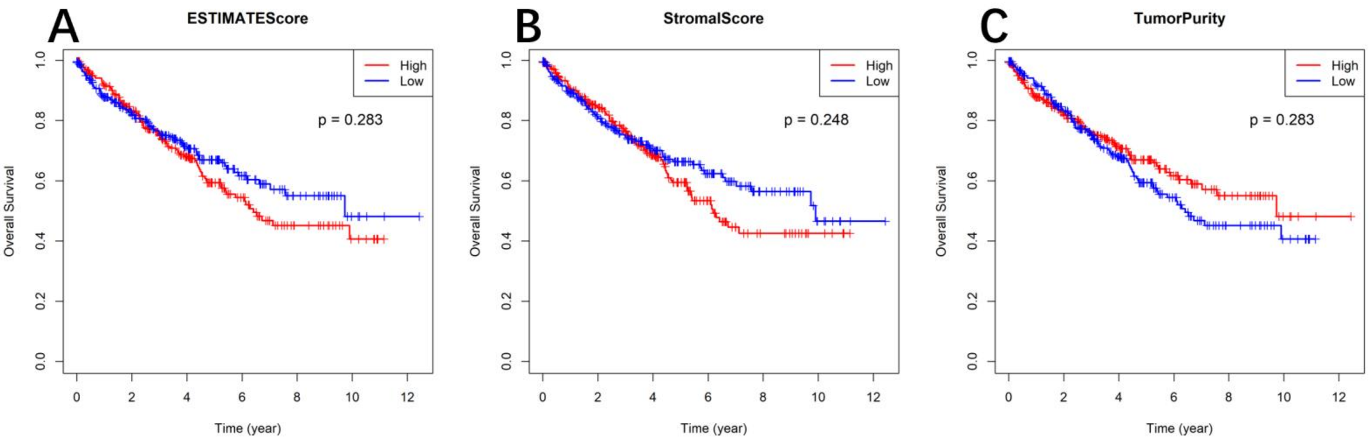

Fig S7. Survival analysis of OS of TICs by ssGSEA scores. (A-W) Survival analysis show the prognosis of of immune cells and immune function in the risk group.

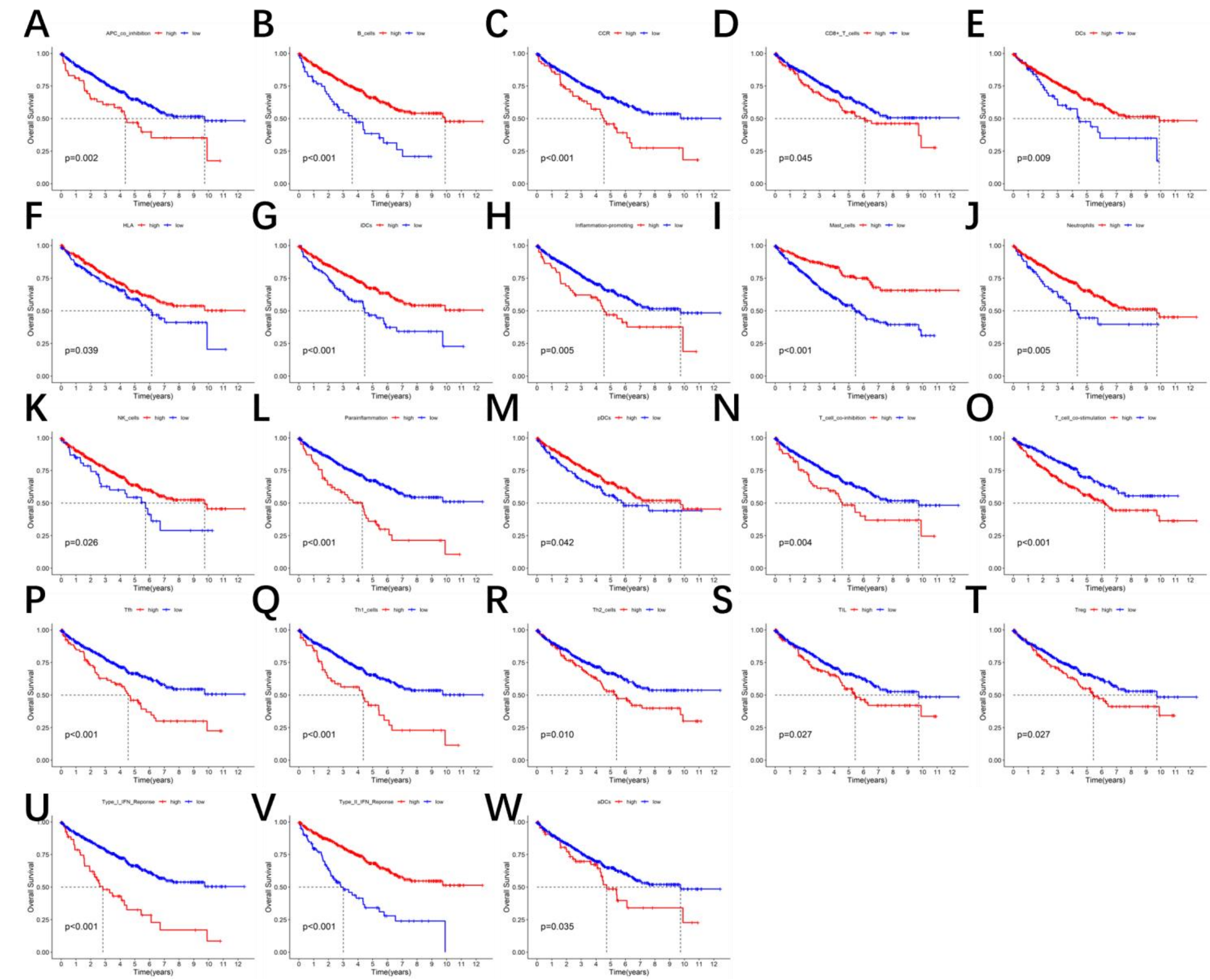

Fig S8. Gene–drug sensitivity analysis based on the CellMiner database. the top 16 drugs with high correlation with gene expression were screened.

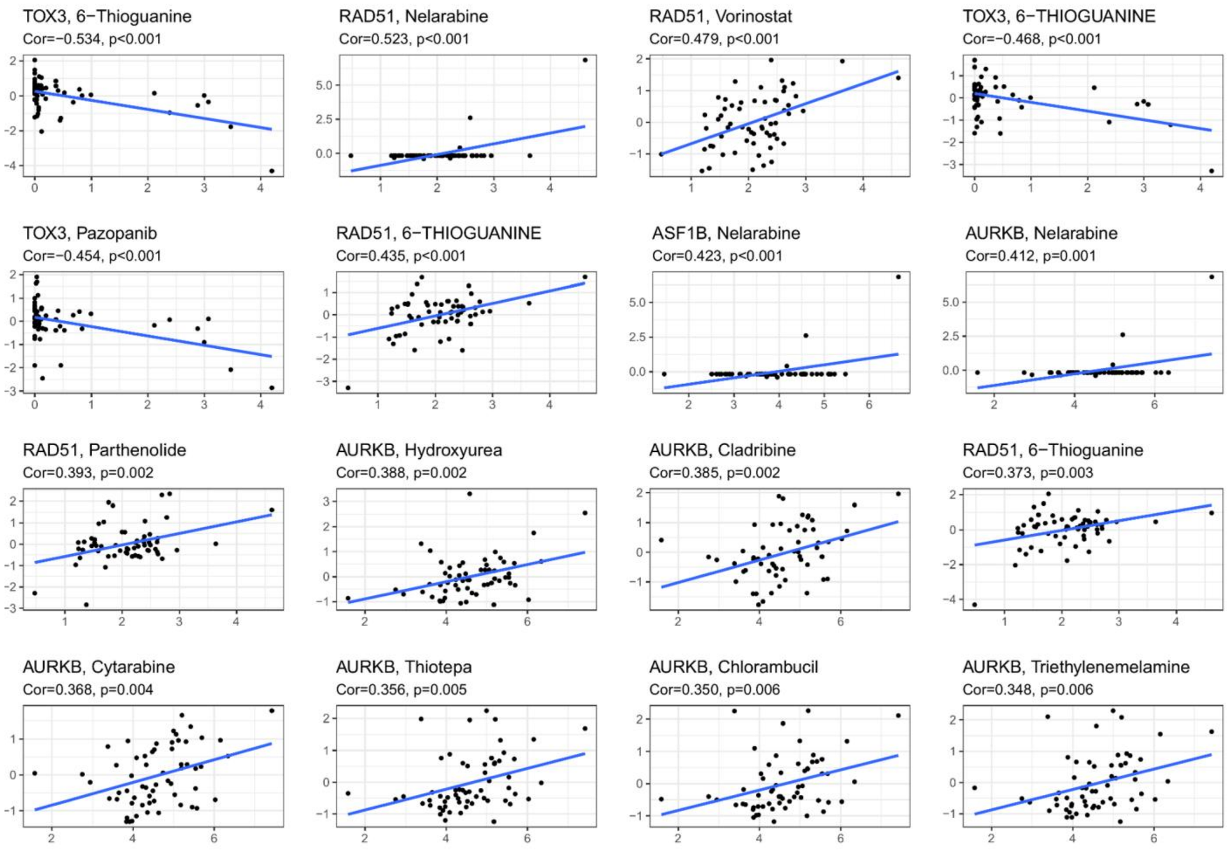

Supplementary Table 1. The sample distribution in training and testing group.

Covariates	Type	Total	Test	Train	Pvalue
Age	<=65	349(65.6%)	171(64.29%)	178(66.92%)	0.584
Age	>65	183(34.4%)	95(35.71%)	88(33.08%)	
Gender	FEMALE	187(35.15%)	99(37.22%)	88(33.08%)	0.3638
Gender	MALE	345(64.85%)	167(62.78%)	178(66.92%)	
Grade	G1	14(2.63%)	4(1.5%)	10(3.76%)	0.321
Grade	G2	228(42.86%)	113(42.48%)	115(43.23%)	
Grade	G3	206(38.72%)	109(40.98%)	97(36.47%)	
Grade	G4	76(14.29%)	36(13.53%)	40(15.04%)	
Grade	unknow	8(1.5%)	4(1.5%)	4(1.5%)	
Stage	Stage I	266(50%)	134(50.38%)	132(49.62%)	0.7009
Stage	Stage II	57(10.71%)	27(10.15%)	30(11.28%)	
Stage	Stage III	123(23.12%)	66(24.81%)	57(21.43%)	
Stage	Stage IV	83(15.6%)	38(14.29%)	45(16.92%)	
Stage	unknow	3(0.56%)	1(0.38%)	2(0.75%)	
T	T1	272(51.13%)	139(52.26%)	133(50%)	0.4143
T	T2	69(12.97%)	32(12.03%)	37(13.91%)	
T	T3	180(33.83%)	92(34.59%)	88(33.08%)	
T	T4	11(2.07%)	3(1.13%)	8(3.01%)	
M	M0	421(79.14%)	214(80.45%)	207(77.82%)	0.3461
M	M1	79(14.85%)	35(13.16%)	44(16.54%)	
M	unknow	32(6.02%)	17(6.39%)	15(5.64%)	
N	N0	240(45.11%)	116(43.61%)	124(46.62%)	0.7225

N	N1	16(3.01%)	9(3.38%)	7(2.63%)	
N	unknow	276(51.88%)	141(53.01%)	135(50.75%)	

Supplementary Table 2. Gsea report for high risk group.

NAME	SIZE	NOM p-val	FDR q-val	FWER p-val	RANK AT MAX
KEGG_HOMOLOGOUS_RECOMBINATION	28	0.002008032	0.17308952	0.151	4735
KEGG_CYTOSOLIC_DNA_SENSING_PATHWAY	55	0.014767933	0.17699097	0.235	8690
KEGG_CYTOKINE_CYTOKINE_RECEPTOR_INTERACTION	264	0.007662835	0.124075696	0.244	9099
KEGG_INTESTINAL_IMMUNE_NETWORK_FOR_IGA_PRODUCTION	46	0.032	0.11329319	0.27	12010
KEGG_PRIMARY_IMMUNODEFICIENCY	35	0.026530612	0.11363912	0.314	10491
KEGG_SYSTEMIC_LUPUS_ERYTHEMATOSUS	137	0.015873017	0.15867662	0.429	20237
KEGG_ASTHMA	28	0.051652893	0.16286005	0.467	11564
KEGG_GLYCOSAMINOGLYCAN_BIOSYNTHESIS_CHONDROITIN_SULFATE	22	0.045454547	0.15962261	0.492	17157
KEGG_AUTOIMMUNE_THYROID_DISEASE	50	0.060606062	0.15085265	0.509	11564
KEGG_HEMATOPOIETIC_CELL_LINEAGE	85	0.07377049	0.232964	0.649	12379
KEGG_PROTEASOME	46	0.09689923	0.21213	0.65	12000
KEGG_P53_SIGNALING_PATHWAY	68	0.074074075	0.21437441	0.684	8033
KEGG_RIBOSOME	88	0.13373253	0.24235079	0.744	9075
KEGG_TYPE_I_DIABETES_MELLITUS	41	0.15631263	0.2734349	0.792	11564
KEGG_ALLOGRAFT_REJECTION	35	0.15368852	0.2590286	0.798	11564
KEGG_JAK_STAT_SIGNALING_PATHWAY	155	0.10973085	0.25036228	0.804	8063
KEGG_NATURAL_KILLER_CELL_MEDIATED_CYTOTOXICITY	132	0.14198783	0.2432014	0.819	9622
KEGG_ALPHA_LINOLENIC_ACID_METABOLISM	19	0.09001957	0.26282638	0.85	8829
KEGG_BASE_EXCISION_REPAIR	35	0.17991632	0.2786253	0.879	16306
KEGG_GRAFT_VERSUS_HOST_DISEASE	37	0.20858896	0.27466264	0.887	13340
KEGG_LEISHMANIA_INFECTION	70	0.23541248	0.31264803	0.924	12093
KEGG_BASAL_CELL_CARCINOMA	55	0.18049793	0.30487078	0.927	13376
KEGG_VIRAL_MYOCARDITIS	68	0.21792261	0.31273344	0.942	11564
KEGG_PYRIMIDINE_METABOLISM	98	0.17782427	0.30853698	0.946	5929
KEGG_ANTIGEN_PROCESSING_AND_PRESENTATION	81	0.23203285	0.306073	0.947	11564
KEGG_NOD_LIKE_RECEPTOR_SIGNALING_PATHWAY	62	0.24476987	0.32034007	0.966	9478
KEGG_PRION_DISEASES	35	0.23622048	0.3257996	0.972	7480
KEGG_GLYCOSAMINOGLYCAN_DEGRADATION	21	0.2074364	0.32820618	0.978	7957
KEGG_HEDGEHOG_SIGNALING_PATHWAY	56	0.23045267	0.32580203	0.98	5206
KEGG_TOLL_LIKE_RECEPTOR_SIGNALING_PATHWAY	102	0.27405858	0.32593873	0.984	7907
KEGG_CELL_CYCLE	125	0.2857143	0.31545204	0.984	9417
KEGG_DNA_REPLICATION	36	0.3125	0.32560855	0.99	4127
KEGG_AMYOTROPHIC_LATERAL_SCLEROSIS_ALS	53	0.26242545	0.36025956	0.995	10963
KEGG_LINOLEIC_ACID_METABOLISM	29	0.26447877	0.35410273	0.995	9073
KEGG_PATHOGENIC_ESCHERICHIA_COLI_INFECTION	56	0.34274194	0.394204	0.999	4109
KEGG_ECM_RECEPTOR_INTERACTION	84	0.33269963	0.38984492	0.999	7685
KEGG_TASTE_TRANSDUCTION	51	0.39503816	0.38417974	0.999	17698
KEGG_GLYCOSAMINOGLYCAN_BIOSYNTHESIS_KERATAN_SULFATE	15	0.36489153	0.3953062	0.999	10593
KEGG_GLYCOSPHINGOLIPID_BIOSYNTHESIS_GANGLIO_SERIES	15	0.39658445	0.43698978	1	7957
KEGG_NOTCH_SIGNALING_PATHWAY	47	0.43035343	0.44232097	1	10353
KEGG_MISMATCH_REPAIR	23	0.47638604	0.45167065	1	4127
KEGG_DRUG_METABOLISM_OTHER_ENZYMES	51	0.57864076	0.5298582	1	5316
KEGG_COMPLEMENT_AND_COAGULATION_CASCADES	69	0.5518591	0.5265336	1	4205
KEGG_OTHER_GLYCAN_DEGRADATION	16	0.5606695	0.5466204	1	7957
KEGG_MATURITY_ONSET_DIABETES_OF_THE_YOUNG	25	0.6132265	0.5554223	1	8834

KEGG_OLFACTORY_TRANSDUCTION	387	0.7148515	0.66813797	1	23828

Supplementary Table 3. Gsea report for low risk group.

NAME	SIZE	NOM p-val	FDR q-val	FWER p-val	RANK AT MAX
KEGG_PROXIMAL_TUBULE_BICARBONATE_RECLAMATION	23	0	0.005306353	0.005	4968
KEGG_TIGHT_JUNCTION	131	0	0.004936591	0.008	5850
KEGG_PROPANOATE_METABOLISM	33	0	0.016845524	0.026	6392
KEGG_VALINE_LEUCINE_AND_ISOLEUCINE_DEGRADATION	44	0	0.018626535	0.036	4310
KEGG_TRYPTOPHAN_METABOLISM	40	0	0.019492475	0.047	7547
KEGG_FATTY_ACID_METABOLISM	42	0.003952569	0.019567857	0.055	8896
KEGG_PEROXISOME	78	0.001960784	0.016772449	0.055	6256
KEGG_BUTANOATE_METABOLISM	34	0.005802708	0.016323563	0.058	8896
KEGG_PYRUVATE_METABOLISM	40	0.003960396	0.015030239	0.058	9178
KEGG_MELANOMA	71	0	0.01402716	0.059	5009
KEGG_SPHINGOLIPID_METABOLISM	39	0	0.017149549	0.08	5510
KEGG_ALDOSTERONE_REGULATED_SODIUM_REABSORPTION	42	0	0.019061638	0.091	2611
KEGG_PROSTATE_CANCER	89	0.001919386	0.022396626	0.108	5116
KEGG_ADIPOCYTOKINE_SIGNALING_PATHWAY	66	0.007905139	0.025151094	0.124	6866
KEGG_INSULIN_SIGNALING_PATHWAY	136	0.001908397	0.02924545	0.143	6894
KEGG_VASOPRESSIN_REGULATED_WATER_REABSORPTION	44	0.003921569	0.02741761	0.143	7958
KEGG_GLYCOSYLPHOSPHATIDYLINOSITOL_GPI_ANCHOR_BIOSYNTHESIS	25	0.009433962	0.026601722	0.144	7486
KEGG_ENDOMETRIAL_CANCER	52	0.001968504	0.026603714	0.15	4371
KEGG_BIOSYNTHESIS_OF_UNSATURATED_FATTY_ACIDS	22	0.001897533	0.026195122	0.153	4032
KEGG_CITRATE_CYCLE_TCA_CYCLE	31	0.00984252	0.027261164	0.16	6823
KEGG_ERBB_SIGNALING_PATHWAY	87	0.003831418	0.026562542	0.162	6750
KEGG_GLYCOLYSIS_GLUCONEOGENESIS	61	0.008080808	0.027753068	0.166	9507
KEGG_ENDOCYTOSIS	181	0.003838772	0.028101055	0.172	7290
KEGG_LONG_TERM_POTENTIATION	70	0.00403225	0.03346125	0.206	7357

		8			
KEGG_NON_SMALL_CELL_LUNG_CANCER	54	0.00952381	0.032725524	0.209	4371
KEGG_TERPENOID_BACKBONE_BIOSYNTHESIS	15	0.001883239	0.032330707	0.21	7931
KEGG_RENAL_CELL_CARCINOMA	70	0.009505703	0.031133274	0.21	5845
KEGG_ADHERENS_JUNCTION	73	0.01192843	0.03431157	0.236	6990
KEGG_LYSINE_DEGRADATION	44	0.023529412	0.037379377	0.253	6392
KEGG_EPITHELIAL_CELL_SIGNALING_IN_HELICOBACTER_PYLORI_INFECTION	68	0.011904762	0.03658923	0.254	6750
KEGG_MTOR_SIGNALING_PATHWAY	52	0.009652509	0.039337058	0.273	5036
KEGG_PPAR_SIGNALING_PATHWAY	69	0.008048289	0.038107775	0.273	9848
KEGG_TYPE_II_DIABETES_MELLITUS	47	0.003802281	0.038063698	0.274	6750
KEGG_O_GLYCAN_BIOSYNTHESIS	30	0.012096774	0.038299896	0.283	6796
KEGG_ONE_CARBON_POOL_BY_FOLATE	17	0.012987013	0.038829815	0.288	4763
KEGG_INOSITOL_PHOSPHATE_METABOLISM	54	0.007766991	0.038888108	0.293	6196
KEGG_LONG_TERM_DEPRESSION	70	0.004065041	0.043099903	0.316	7355
KEGG_NEUROTROPHIN_SIGNALING_PATHWAY	126	0.018832391	0.042031024	0.316	7509
KEGG_PANCREATIC_CANCER	70	0.023346303	0.042215247	0.322	5664
KEGG_BETA_ALANINE_METABOLISM	22	0.026266417	0.044860944	0.334	3959
KEGG_TGF_BETA_SIGNALING_PATHWAY	86	0.03929273	0.046178035	0.345	7827
KEGG_REGULATION_OF_AUTOPHAGY	35	0.007889546	0.045277346	0.345	4415
KEGG_GLIOMA	65	0.019011406	0.047835875	0.364	5207
KEGG_THYROID_CANCER	29	0.013513514	0.047727205	0.368	4371
KEGG_RENIN_ANGIOTENSIN_SYSTEM	17	0.015968064	0.04946566	0.376	7566
KEGG_GLYCINE_SERINE_AND_THREONINE_METABOLISM	31	0.024809161	0.052302316	0.394	8907
KEGG_COLORECTAL_CANCER	62	0.02534113	0.05246429	0.396	4874
KEGG_HISTIDINE_METABOLISM	29	0.03809524	0.05258153	0.398	7547
KEGG_GLYCEROLIPID_METABOLISM	49	0.013972056	0.052183457	0.401	8896
KEGG_GNRH_SIGNALING_PATHWAY	101	0.011516315	0.05834629	0.431	7905
KEGG_CHRONIC_MYELOID_LEUKEMIA	73	0.03244275	0.058108516	0.433	4874
KEGG_WNT_SIGNALING_PATHWAY	151	0.02004008	0.06116718	0.455	7026

KEGG_PANTOTHENATE_AND_COA_BIOSYNTHESIS	16	0.024761904	0.06241688	0.462	8988
KEGG_NITROGEN_METABOLISM	23	0.006237006	0.06673787	0.492	12284
KEGG_BLADDER_CANCER	42	0.026871402	0.071934536	0.529	4692
KEGG_UBIQUITIN_MEDIATED_PROTEOLYSIS	135	0.060311284	0.074126355	0.543	6818
KEGG_ETHER_LIPID_METABOLISM	33	0.00984252	0.07352282	0.545	2930
KEGG_REGULATION_OF_ACTIN_CYTOSKELETON	213	0.03508772	0.07306924	0.547	5845
KEGG_HUNTINGTONS_DISEASE	182	0.067961164	0.07188954	0.547	8374
KEGG_PHOSPHATIDYLINOSITOL_SIGNALING_SYSTEM	76	0.038910504	0.07275024	0.554	6965
KEGG_GLYOXYLATE_AND_DICARBOXYLATE_METABOLISM	16	0.044	0.07157659	0.554	4247
KEGG_MAPK_SIGNALING_PATHWAY	267	0.013565891	0.07326684	0.562	7646
KEGG_PATHWAYS_IN_CANCER	325	0.03522505	0.07623148	0.579	7026
KEGG_DORSO_VENTRAL_AXIS_FORMATION	24	0.024952015	0.07747194	0.59	7802
KEGG_MELANOGENESIS	101	0.0331384	0.08134885	0.599	5207
KEGG_VASCULAR_SMOOTH_MUSCLE_CONTRACTION	115	0.062992126	0.08086481	0.603	6444
KEGG_OOCYTE_MEIOSIS	113	0.052941177	0.08309854	0.616	5494
KEGG_STEROID_BIOSYNTHESIS	17	0.054054055	0.09021729	0.65	8288
KEGG_ALZHEIMERS_DISEASE	166	0.087128714	0.090040766	0.653	8374
KEGG_LEUKOCYTE_TRANSENDOTHELIAL_MIGRATION	116	0.0499002	0.08953896	0.653	4803
KEGG_SELENOAMINO_ACID_METABOLISM	26	0.059961315	0.08957994	0.658	5811
KEGG_CYSTEINE_AND_METHIONINE_METABOLISM	34	0.048780486	0.09375157	0.67	5103
KEGG_VIBRIO_CHOLERAE_INFECTION	54	0.06903765	0.096619934	0.682	6293
KEGG_N_GLYCAN_BIOSYNTHESIS	46	0.09542744	0.096134916	0.684	6245
KEGG_VEGF_SIGNALING_PATHWAY	76	0.017274473	0.09623961	0.685	4371
KEGG_PROGESTERONE_MEDIATED_OOCYTE_MATURATION	85	0.07183365	0.09583247	0.685	4671
KEGG_GAP_JUNCTION	90	0.061143983	0.09618248	0.688	6367
KEGG_CALCIUM_SIGNALING_PATHWAY	178	0.03550296	0.09643015	0.693	7356
KEGG_FC_EPSILON_RI_SIGNALING_PATHWAY	79	0.04789272	0.09566142	0.696	7735
KEGG_ARGININE_AND_PROLINE_METABOLISM	54	0.069327734	0.09661675	0.702	8896
KEGG_FOCAL_ADHESION	199	0.11306043	0.09820475	0.708	5928
KEGG_ACUTE_MYELOID_LEUKEMIA	57	0.07869482	0.10436691	0.732	6109
KEGG_AXON_GUIDANCE	129	0.1009901	0.10956136	0.752	6230
KEGG_PROTEIN_EXPORT	24	0.117529884	0.11130401	0.761	5755
KEGG_METABOLISM_OF_XENOBIOTICS_BY_CYTOCHROME_P450	69	0.07172131	0.12030928	0.789	9203

KEGG_LYSOSOME	121	0.119521916	0.1205886	0.794	7497
KEGG_DRUG_METABOLISM_CYTOCHROME_P450	71	0.06418219	0.12019187	0.796	9203
KEGG_RETINOL_METABOLISM	64	0.048879836	0.12948567	0.819	9224
KEGG_GLYCEROPHOSPHOLIPID_METABOLISM	76	0.04892368	0.12869921	0.819	4782
KEGG_ARRHYTHMOGENIC_RIGHT_VENTRICULAR_CARDIOMYOPATHY_ARVC	74	0.11293635	0.13141656	0.824	5294
KEGG_SMALL_CELL_LUNG_CANCER	84	0.14003944	0.1391426	0.837	4780
KEGG_OXIDATIVE_PHOSPHORYLATION	132	0.16973415	0.14823072	0.853	7788
KEGG_RNA_DEGRADATION	59	0.16666667	0.15346989	0.862	6250
KEGG_APOPTOSIS	87	0.14591439	0.15362594	0.863	4730
KEGG_PHENYLALANINE_METABOLISM	18	0.114164904	0.16168813	0.881	2976
KEGG_TYROSINE_METABOLISM	42	0.111561865	0.16798459	0.895	9311
KEGG_ASCORBATE_AND_ALDARATE_METABOLISM	25	0.15071283	0.17564434	0.902	8911
KEGG_PARKINSONS_DISEASE	130	0.22709164	0.18142754	0.913	8031
KEGG_B_CELL_RECEPTOR_SIGNALING_PATHWAY	75	0.17716536	0.18175195	0.914	4371
KEGG_GLYCOSPHINGOLIPID_BIOSYNTHESIS_LACTO_AND_NEOLACTO_SERIES	26	0.12008282	0.18658185	0.918	7394
KEGG_SNARE_INTERACTIONS_IN_VESICULAR_TRANSPORT	38	0.19428572	0.21116482	0.944	5206
KEGG_ALANINE_ASPARTATE_AND_GLUTAMATE_METABOLISM	32	0.20114942	0.21146297	0.947	8699
KEGG_AMINOACYL_TRNA_BIOSYNTHESIS	41	0.25	0.21311522	0.95	7397
KEGG_PURINE_METABOLISM	159	0.16977613	0.22201625	0.958	8367
KEGG_FRUCTOSE_AND_MANNOSE_METABOLISM	34	0.20380953	0.23263326	0.964	6820
KEGG_NICOTINATE_AND_NICOTINAMIDE_METABOLISM	24	0.18846154	0.23123075	0.964	8137
KEGG_RIG_I_LIKE_RECEPTOR_SIGNALING_PATHWAY	71	0.2109375	0.23957992	0.968	8601
KEGG_PENTOSE_AND_GLUCURONATE_INTERCONVERSIONS	28	0.20754717	0.24708135	0.97	8911
KEGG_FC_GAMMA_R_MEDIATED_PHAGOCYTOSIS	96	0.26129666	0.24698295	0.971	3442
KEGG_STARCH_AND_SUCROSE_METABOLISM	51	0.22986248	0.2585638	0.976	9129
KEGG_GLYCOSAMINOGLYCAN_BIOSYNTHESIS_HEPARAN_SULFATE	26	0.22845691	0.2575389	0.977	4926
KEGG_ABC_TRANSPORTERS	44	0.24031007	0.26361626	0.977	9385
KEGG_NUCLEOTIDE_EXCISION_REPAIR	44	0.28624535	0.2645633	0.979	7524
KEGG_PORPHYRIN_AND_CHLOROPHYLL_METABOLISM	41	0.24193548	0.26305774	0.979	9459
KEGG_T_CELL_RECEPTOR_SIGNALING_PATHWAY	108	0.30528376	0.26706746	0.98	5845
KEGG_HYPERTROPHIC_CARDIOMYOPATHY_HCM	83	0.25720164	0.2692772	0.981	6268
KEGG_GLUTATHIONE_METABOLISM	49	0.27952754	0.2799476	0.983	6906
KEGG_CELL_ADHESION_MOLECULES_CAMS	131	0.28657314	0.27921999	0.984	6230
KEGG_DILATED_CARDIOMYOPATHY	90	0.26052105	0.28161663	0.987	5294
KEGG_PRIMARY_BILE_ACID_BIOSYNTHESIS	16	0.28806585	0.28241718	0.988	9389
KEGG_GALACTOSE_METABOLISM	25	0.2990099	0.28808793	0.99	9129
KEGG_CHEMOKINE_SIGNALING_PATHWAY	188	0.34251967	0.3053063	0.994	4656
KEGG_PENTOSE_PHOSPHATE_PATHWAY	27	0.32462686	0.3358142	0.998	9507
KEGG_BASAL_TRANSCRIPTION_FACTORS	35	0.3847619	0.34086972	0.998	9622
KEGG_SPLICEOSOME	127	0.3815029	0.3435374	0.998	5791
KEGG_AMINO_SUGAR_AND_NUCLEOTIDE_SUGAR_METABOLISM	44	0.39029127	0.36595845	0.999	4599
KEGG_STEROID_HORMONE_BIOSYNTHESIS	55	0.5042194	0.40612656	1	9072
KEGG_NEUROACTIVE_LIGAND_RECEPTOR_INTERACTION	272	0.4623218	0.41135326	1	7975
KEGG_RNA_POLYMERASE	29	0.4728972	0.42425677	1	7113
KEGG_RIBOFLAVIN_METABOLISM	16	0.46804512	0.43047237	1	5118
KEGG_ARACHIDONIC_ACID_METABOLISM	58	0.5748988	0.48109233	1	4151
KEGG_CARDIAC_MUSCLE_CONTRACTION	79	0.54219407	0.4894782	1	8374

Supplementary Table 4. Correlations between risk score and immune cell infiltrations by following software: XCELL; TIMER; QUANTISEQ; MCPCOUNTER; EPIC; CIBERSORT-ABS and CIBERSORT.

immune	cor	pvalue
T cell CD8+_TIMER	0.11750696	0.006660802
Neutrophil_TIMER	0.150018787	0.000517117
Myeloid dendritic cell_TIMER	0.167590856	0.000102788
B cell naive_CIBERSORT	-0.234216012	4.61E-08
B cell memory_CIBERSORT	0.18312815	2.14E-05
B cell plasma_CIBERSORT	-0.117857539	0.006498727
T cell CD8+_CIBERSORT	0.239790655	2.15E-08
T cell CD4+ memory resting_CIBERSORT	-0.134380584	0.001894349
T cell CD4+ memory activated_CIBERSORT	0.158496838	0.000242194
T cell follicular helper_CIBERSORT	0.297548464	2.45E-12
T cell regulatory (Tregs)_CIBERSORT	0.364625497	3.58E-18
T cell gamma delta_CIBERSORT	0.107883865	0.012782497
NK cell resting_CIBERSORT	-0.173605604	5.69E-05
Monocyte_CIBERSORT	-0.15504061	0.000331508
Macrophage M0_CIBERSORT	0.122647855	0.004612254
Macrophage M2_CIBERSORT	-0.145985434	0.000731845
Myeloid dendritic cell resting_CIBERSORT	0.101185838	0.01957644
Myeloid dendritic cell activated_CIBERSORT	-0.142749412	0.000960966
Mast cell activated_CIBERSORT	-0.217462519	4.09E-07
Eosinophil_CIBERSORT	-0.105893296	0.014542712
B cell naive_CIBERSORT-ABS	-0.189171129	1.12E-05
B cell memory_CIBERSORT-ABS	0.184236131	1.90E-05
T cell CD8+_CIBERSORT-ABS	0.244182526	1.16E-08
T cell CD4+ memory activated_CIBERSORT-ABS	0.158977929	0.000231719
T cell follicular helper_CIBERSORT-ABS	0.319609267	4.24E-14
T cell regulatory (Tregs)_CIBERSORT-ABS	0.394758069	2.77E-21
T cell gamma delta_CIBERSORT-ABS	0.118394454	0.006257359
NK cell resting_CIBERSORT-ABS	-0.163085999	0.000158046
NK cell activated_CIBERSORT-ABS	0.147720134	0.000630975
Macrophage M0_CIBERSORT-ABS	0.128837004	0.002910193
Macrophage M1_CIBERSORT-ABS	0.126638992	0.003435036
Myeloid dendritic cell resting_CIBERSORT-ABS	0.107993489	0.012691267
Myeloid dendritic cell activated_CIBERSORT-ABS	-0.139291717	0.001277713
Mast cell activated_CIBERSORT-ABS	-0.188881593	1.16E-05
Macrophage M1_QUANTISEQ	0.390838563	7.34E-21
Macrophage M2_QUANTISEQ	0.0869857	0.044918237
Monocyte_QUANTISEQ	0.145604655	0.000755899
Neutrophil_QUANTISEQ	-0.283531041	2.71E-11
NK cell_QUANTISEQ	-0.171246183	7.19E-05
T cell CD4+ (non-regulatory)_QUANTISEQ	-0.257846016	1.58E-09
T cell CD8+_QUANTISEQ	0.317414624	6.44E-14
Myeloid dendritic cell_QUANTISEQ	-0.191126865	9.04E-06
T cell CD8+_MCPCOUNTER	0.259807608	1.18E-09
cytotoxicity score_MCPCOUNTER	0.236898298	3.20E-08
Monocyte_MCPCOUNTER	0.141544056	0.001062064
Macrophage/Monocyte_MCPCOUNTER	0.141544056	0.001062064

Neutrophil_MCPCOUNTER	-0.345164635	2.49E-16
Endothelial cell_MCPCOUNTER	-0.271292907	1.99E-10
Cancer associated fibroblast_MCPCOUNTER	0.223546788	1.89E-07
Myeloid dendritic cell activated_XCELL	0.311085199	2.12E-13
B cell_XCELL	0.279183401	5.56E-11
T cell CD4+ naive_XCELL	0.156960224	0.00027869
T cell CD4+ effector memory_XCELL	0.275358334	1.04E-10
T cell CD8+_XCELL	0.257361654	1.70E-09
T cell CD8+ central memory_XCELL	0.268839319	2.93E-10
T cell CD8+ effector memory_XCELL	0.288989006	1.08E-11
Class-switched memory B cell_XCELL	0.18268363	2.24E-05
Common lymphoid progenitor_XCELL	-0.206549808	1.55E-06
Myeloid dendritic cell_XCELL	0.131985959	0.002284856
Endothelial cell_XCELL	-0.208518422	1.22E-06
Eosinophil_XCELL	-0.099593628	0.02159307
Granulocyte-monocyte progenitor_XCELL	-0.150382739	0.000500948
Hematopoietic stem cell_XCELL	-0.341626937	5.22E-16
Macrophage_XCELL	0.222679115	2.11E-07
Macrophage M1_XCELL	0.307899694	3.81E-13
Macrophage M2_XCELL	0.161019126	0.000191819
B cell memory_XCELL	0.086905382	0.04511715
Monocyte_XCELL	0.291302824	7.27E-12
B cell naive_XCELL	0.184402035	1.87E-05
T cell NK_XCELL	0.470495619	1.17E-30
Plasmacytoid dendritic cell_XCELL	0.283852617	2.57E-11
B cell plasma_XCELL	0.224875307	1.59E-07
T cell CD4+ Th1_XCELL	0.431872306	1.39E-25
T cell CD4+ Th2_XCELL	0.294730855	4.01E-12
immune score_XCELL	0.321049293	3.21E-14
stroma score_XCELL	-0.127360816	0.003253894
microenvironment score_XCELL	0.233585047	5.02E-08
B cell_EPIC	-0.187685374	1.32E-05
Cancer associated fibroblast_EPIC	0.2856895	1.89E-11
T cell CD4+_EPIC	-0.412096727	3.17E-23
T cell CD8+_EPIC	-0.122690863	0.004597832
Endothelial cell_EPIC	-0.309201328	3.00E-13
Macrophage_EPIC	0.343850134	3.28E-16
NK cell_EPIC	0.300818319	1.37E-12
uncharacterized cell_EPIC	0.209486083	1.09E-06
